# Next generation sequencing identifies novel disease-associated BEST1 mutations in Bestrophinopathy patients

**DOI:** 10.1038/s41598-018-27951-8

**Published:** 2018-07-05

**Authors:** Thong T. Nguyen, B. Poornachandra, Anshuman Verma, Ruchir A. Mehta, Sameer Phalke, Rajani Battu, Vedam L. Ramprasad, Andrew S. Peterson, Arkasubhra Ghosh, Somasekar Seshagiri

**Affiliations:** 1 GROW Research Laboratory, Narayana Nethralaya Foundation, Bangalore, India; 2 Department of Molecular Biology, Genentech Inc, San Francisco, USA; 3 Medgenome Inc., Bangalore, India; 4 Retina Department, Narayana Nethralaya, Bangalore, India

## Abstract

Bestinopathies are a spectrum of retinal disorders associated with mutations in *BEST1* including autosomal recessive bestrophinopathy (ARB) and autosomal dominant Best vitelliform macular dystrophy (BVMD). We applied whole-exome sequencing on four unrelated Indian families comprising eight affected and twelve unaffected individuals. We identified five mutations in *BEST1*, including p.Tyr131Cys in family A, p.Arg150Pro in family B, p.Arg47His and p.Val216Ile in family C and p.Thr91Ile in family D. Among these, p.Tyr131Cys, p.Arg150Pro and p.Val216Ile have not been previously reported. Further, the inheritance pattern of *BEST1* mutations in the families confirmed the diagnosis of ARB in probands in families A, B and C, while the inheritance of heterozygous *BEST1* mutation in family D (p.Thr91Ile) was suggestive of BVMD. Interestingly, the ARB families A and B carry homozygous mutations while family C was a compound heterozygote with a mutation in an alternate *BEST1* transcript isoform, highlighting a role for alternate *BEST1* transcripts in bestrophinopathy. In the BVMD family D, the heterozygous *BEST1* mutation found in the proband was also found in the asymptomatic parent, suggesting an incomplete penetrance and/or the presence of additional genetic modifiers. Our report expands the list of pathogenic *BEST1* genotypes and the associated clinical diagnosis.

## Introduction

Mutations in *BEST1* gene have been described in a variety of ocular disease phenotypes including autosomal recessive bestrophinopathy (ARB, MIM 611809)^1,2^, Best vitelliform macular dystrophy (MIM 153700)^3,4^, autosomal dominant vitreoretinochoroidopathy (MIM 193220)^5,6^, autosomal dominant microcornea, rod-cone dystrophy, early-onset cataract posterior staphyloma syndrome and retinitis pigmentosa (MIM 613194)^7,8^. The BEST1 (bestrophin-1) protein is expressed in the basolateral plasma membrane of the retinal pigment epithelium (RPE) where it regulates multiple functions essential for normal vision^7,9^. It primarily functions as a calcium-activated chloride channel^7,9,10^. A role for bestrophin-1 in normal ocular development has been proposed, though its precise role in this context is not fully understood^7^.

Best vitelliform macular dystrophy (BVMD) is an autosomal dominant syndrome associated with *BEST1* mutations^10^. BVMD primarily affects the macula and is characterized by yellowish, vitelliform or egg-yolk-like lesions with considerable morphologic variations based on the stage of the disease^11,12^. Electrophysiological characteristics in BVMD patients include normal full-field electroretinography (ERG), with a marked decrease or absence of light rise in electro-oculography (EOG)^13^. Early onset choroidal neovascular membranes in patients with BVMD^14^, often occur before vitelliruption and it responds to intravitreal therapies. Autosomal recessive bestrophinopathy (ARB) (MIM 611809) is another condition associated with mutations in the *BEST1* gene^15^. ARB usually manifests in the first two decades of life, but may become symptomatic as late as the fifth decade^2,15,16^. Clinical presentation of ARB is distinct from BVMD^17^ and is characterized by central visual loss with typical hyperopic conditions, sub-retinal lipofuscin deposits that are predominantly outside the macula, absence of light rise in EOG, reduced ERG, accumulation of fluid within and/or beneath the neurosensory retina and development of angle-closure glaucoma^17,18^. ARB may manifest as the result of a total absence (null phenotype) of functional bestrophin-1 protein in the RPE^15,19^, improper localization to the cell membrane with intact anion channel activity^20^ or lack of channel activity specifically^2^. Among the roughly 270 mutations^21^ reported in *BEST1* thus far, only about 40 compound heterozygous and homozygous mutations are associated with ARB^15,17,18,22^. In this study, we analyzed four Indian families with clinically diagnosed bestrophinopathy using whole exome sequencing and identified novel *BEST1* mutations.

## Materials and Methods

### Study cohort

The prospective study was approved by the Narayana Nethralaya Institutional Review Board and was performed as per institutional ethics guidelines and in accordance with the tenets of the Declaration of Helsinki. Subjects were recruited for the study after obtaining informed written consent either from the patient or the guardian and family members. A total of eight patients with Bestrophinopathy from four unrelated families from southern India were investigated. Age at the time presentation ranged between 11–26 years with mean age of 20 years.

### Clinical examination

Detailed medical history was obtained, followed by clinical examination including best-corrected Snellen visual acuity (BCVA), slit-lamp examination, gonioscopy, indirect ophthalmoscopy and fundus photography. Fundus autofluorescence (FAF) imaging with a confocal scanning laser ophthalmoscope (Spectralis, Heidelberg Engineering, Heidelberg, Germany) in all eight patients and selected family members was performed. Spectral domain optical coherence tomography (SD OCT; Spectralis, Heidelberg Engineering, Heidelberg, Germany) was also performed simultaneously in these patients. Two of the eight patients underwent fundus fluorescein angiography (FFA). Electrophysiologic examinations were conducted according to the standards given by the International Society of Clinical Electrophysiology in Vision^23,24^. Viking 5.0 Ganzfeld dome (Nicolet Biomedical Instruments, Madison, Wisconsin, USA) with a light-emitting diode for light stimulation was used for both electro-oculography and full-field electroretinography.

### DNA isolation, exome library preparation and sequencing

DNA was isolated from whole blood using QIAamp DNA Blood Mini Kit (Qiagen, CA, US; cat no. 51104). Exome library was performed using Agilent SureSelect (Santa Clara, CA, US) Human All Exome kit v5 (50 Mb). The library was sequenced on Illumina HiSeq. 4000 (Illumina, CA, US) to obtain 2 × 75 bp paired end reads. An average exome coverage of 71x (range 45–94x) with 81% of the bases over 20x (range 76–91) was obtained.

### RNA isolation, sequencing and analysis

RNA was isolated from a healthy donor retina sample using RNeasy Mini Kit (Qiagen; catalogue no 74104). About 0.5 μg of total RNA was used to generate RNA-seq library using TruSeq RNA Sample Preparation kit (Illumina). The library was sequenced on HiSeq2500 to obtain 166,513,102 paired-end (2 × 75 bp) reads. RNA-seq reads were mapped to the human reference genome version GRCh37 using GSNAP^25^. Analysis of BEST1 transcript isoforms was performed using the SGSeq software package (version 1.4.0)^26^ with gene annotation from Ensembl (release 75).

### Variant calling and annotation

Whole exome sequencing data was processed using GATK’s best practices workflow for DNA-Seq^27^. Briefly, the raw sequencing reads were aligned to the human reference genome version GRCh37 using BWA-MEM (version 0.7.10)^28^. Picard tools (version 1.126) were used for removing duplicate reads. Indel realignment and base quality score recalibration were performed using GATK (version 3) to improve alignment accuracy and recalibrate base quality^29^. HaplotypeCaller (in reference confidence model) was applied for each sample to generate gVCF files. The resulting gVCF files from all samples were used for joint variant calling using GenotypeGVCFs walker. Variant Quality Score Recalibration (VQSR) was carried out to estimate the confidence of called variants. Variant annotation was carried out using SnpEff program (version 4.2)^30^.

### Variant filtering and analysis

To identity candidate nucleotide variants, we applied a filtering strategy. Those variants which were present at >0.2% in either the 1000 Genome Project^31^ or NHLBI-EVS Exome Sequencing Project^32^ or ExAC database^33^ were filtered out. Protein-altering or potentially protein-altering variants that follow recessive or dominant modes of inheritance were selected for further analysis. Given that *BEST1* is a well-established Bestrophinopathy gene, all nucleotide variants present in *BEST1* were additionally reviewed. The genes and corresponding mutations that qualified these filtering criteria were investigated to determine their significance and relevance in Bestrophinopathy. Genes that were associated with an ocular disease phenotype, as reported on RetNet (https://sph.uth.edu/retnet/), were used for further investigation. Read evidence of the final list of nucleotide variants was manually inspected using Integrative Genomics Viewer (IGV)^34^ to detect sequencing artifacts.

### Ethics approval and consent to participate

Written informed consent was obtained from all the study participants. Consent from the parents or legal guardian was obtained wherever samples from minors were included in the study. The study was approved by the Narayana Nethralaya Institutional Review Board and was performed as per institutional ethics guidelines and in accordance with the tenets of the Declaration of Helsinki.

### Availability of data and materials

Filtered variant data is available as a supplementary table. Genotype data for patients consenting to share it for research use shall be made available upon request.

## Results

### Patients and Clinical Characteristics

**Figure 1:**
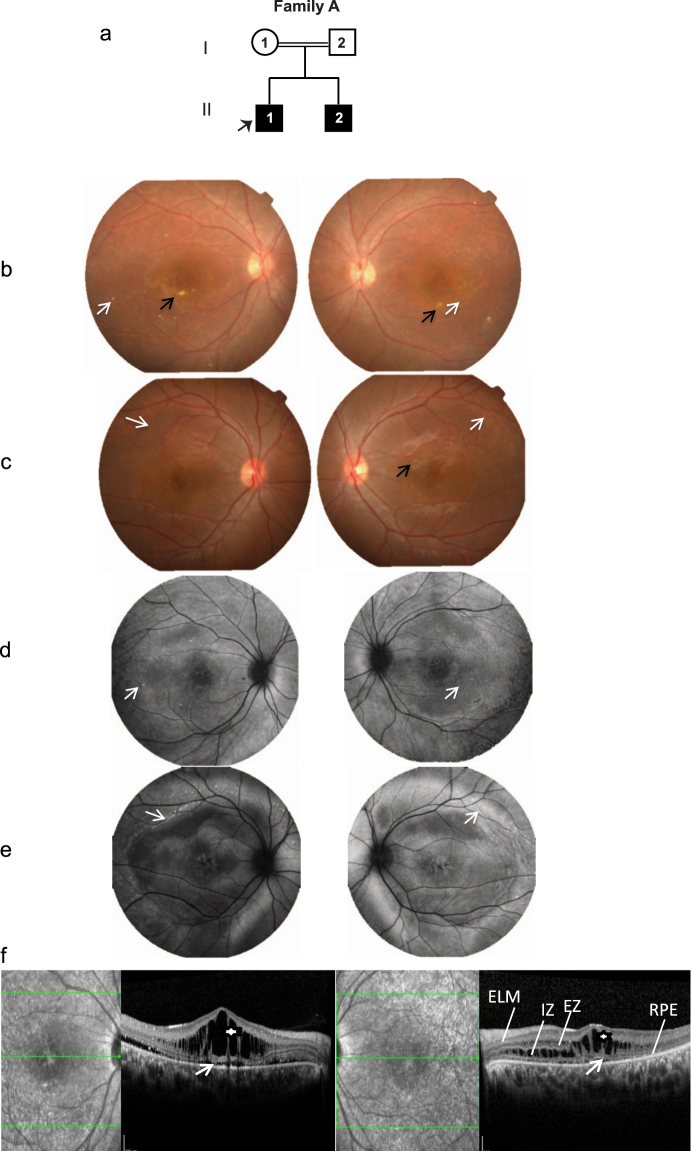
Family A. (**a**) Pedigree of the family with two affected members. The proband is marked with an arrow. (**b**,**c**) Colored fundus photographs of right and left eye of proband (II.1) and affected sibling (II.2) respectively, showing focal areas of subretinal fibrosis (black arrow) with very sparse vitelliform deposit marked by white arrow. (**d**,**e**) Fundus autofluorescence images of proband (II.1) and affected sibling (II.2) respectively, showing focal dot like areas of increased autofluorescence (white arrow) corresponding to the areas of vitelliform deposit seen in fundus photography. (**f**) Optical coherence tomography (OCT) images of the proband showing intra retinal cystoid and schitic changes (asterisk) with minimal sub retinal fluid (white arrow). The sub retinal area is marked with the retinal layers as RPE = Retinal Pigment Epithelium, IZ = Interdigitation Zone, EZ = Ellipsoid Zone and ELM = External Limiting Membrane.

**Figure 2:**
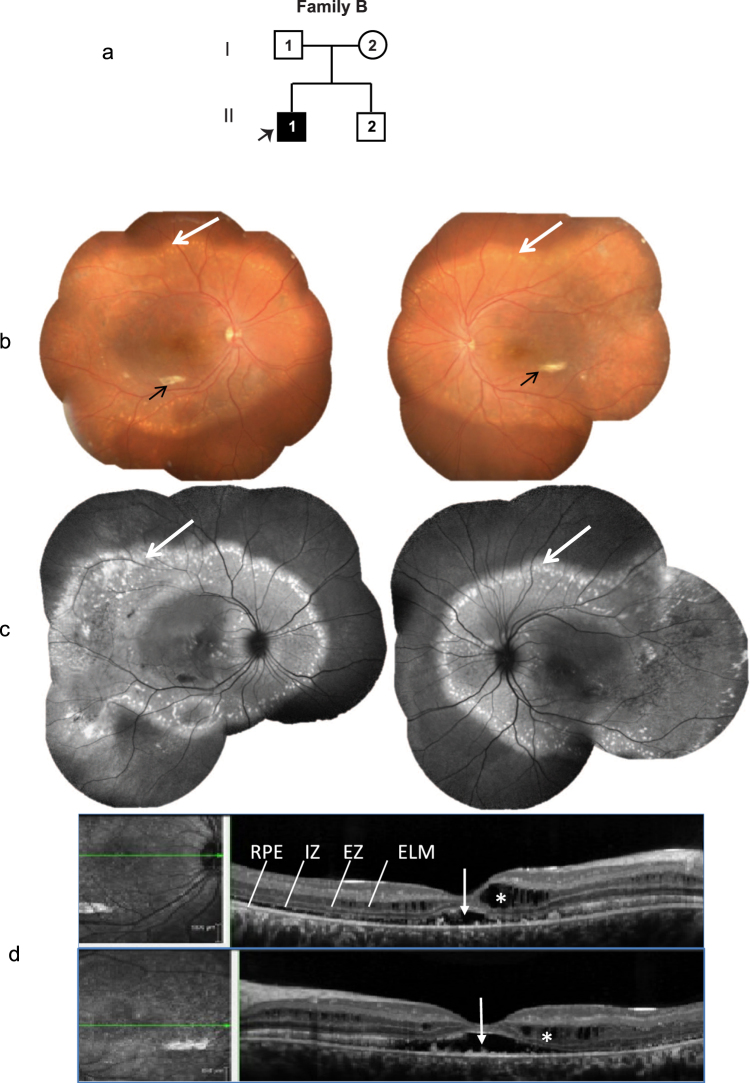
Family B. (**a**) Pedigree of the family with one affected subject (II.1; indicated by arrow). (**b**) Colored fundus photographs of the right and left eyes of proband demonstrating focal areas of sub-retinal fibrosis (black arrow) and extensive areas of sub-retinal vitelliform deposits across the arcade and nasal to the disk (white arrow). (**c**) Corresponding fundus autofluorescence (FAF) images of right and left eyes of the proband highlight the vitelliform deposits (white arrows). (**d**) OCT images of right and left eyes of proband showing sub retinal (white arrow) and intra retinal (white asterisk) changes with fluid accumulation.

**Figure 3:**
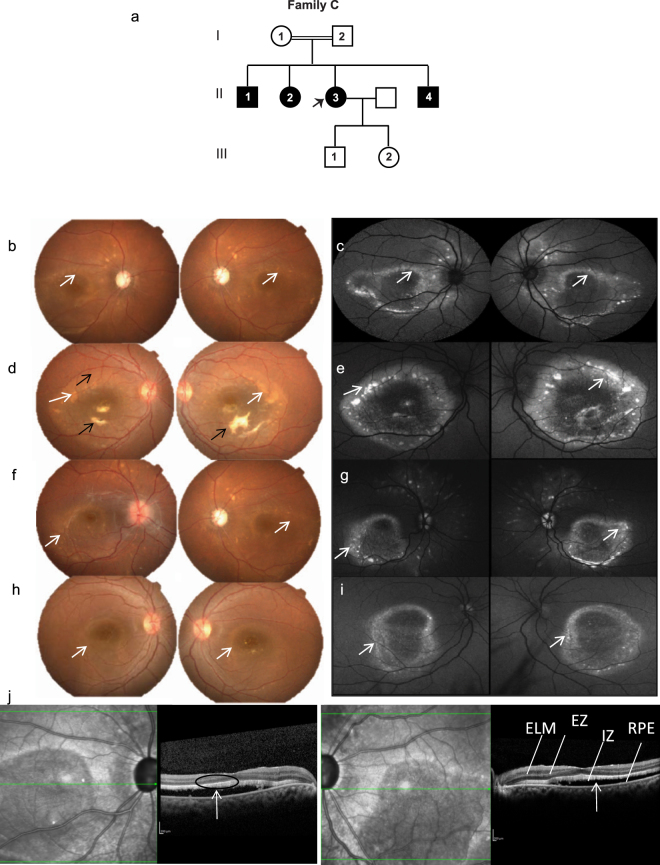
Family C. (**a**) Pedigree of the family with four affected members. The proband is marked with an arrow. Colored fundus photographs (b, d, f and h) of the proband (II.3) (b, c), affected siblings (brother II.1 (**d**,**e**), brother II.4 (**f**,**g**), sister (II.2 (**h**,**i**) show yellowish white sub-retinal deposits (white arrows). Fundus autofluoresence (c, e, g and i) of the proband (II.3) (**b**,**c**), affected siblings (brother II.1 (**d**,**e**), brother II.4 (**f**,**g**), sister (II.2 (**h**,**i**) show hyper autoflourescence (white arrows) mainly concentrated around posterior pole. A localised area of subretinal fibrosis (black arrow; **c**) is observed in affected sibling II.1. (**j**) OCT of right and left eyes of proband show outer segment elongation (black circle) with sub retinal changes and subretinal fluid (white arrow).

**Figure 4:**
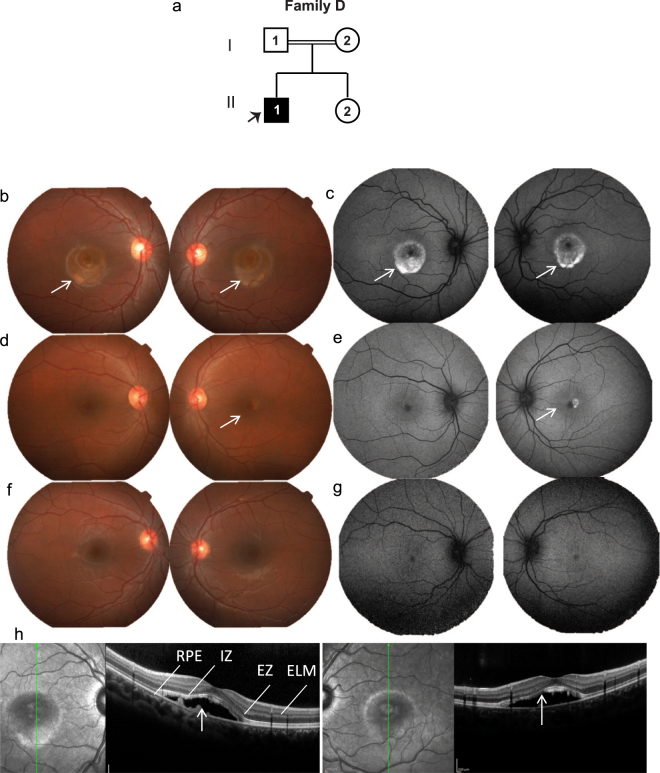
Family D. (**a**) Pedigree chart of family with one affected subject indicated by black arrow. Colored fundus photographs of right and left eyes of proband (**b**), father (**d**) and sister (**f**). Corresponding FAF images of right and left eyes of proband (**c**), father (**e**) and sister (**g**). Proband (**b**), shows prominent yellowish vitelliform deposits in macula giving pseudohypopyon appearance, better highlighted in FAF (**c**). Proband’s father (**d**) shows a normal fundus in the right eye but left eye had focal vitelliform lesion (white arrow) better highlighted in FAF (white arrow) (**e**). Sister (**f**,**g**) did not show abnormal retinal features. (**h**) OCT of the proband shows sub-retinal deposits with sub-retinal fluid in both eyes (white arrows).

Twenty individuals, including eight affected and twelve unaffected members from four families, were selected for the study (Figs 1–4; Table 1).

**Table 1:** Summary of clinical characteristics and genetic findings.

Patient	Age	Sex	CDVA	OCT features	ERG	EOG	Mutation^*^
**FAMILY A**							
Proband (II.1)	21	Male	20/60	Cystoid and schitic changes in macula	Rod-cone dysfunction	Absent light peak	p.Tyr131Cys (hom)
Sibling (II.2)	24	Male	20/60	Cystoid and schitic changes in macula	Rod-cone dysfunction	Absent light peak	p.Tyr131Cys (hom)
**FAMILY B**							
Proband (II.1)	24	Male	20/40, 20/30	Schitic changes with subretinal fluid	Rod-cone dysfunction	Absent light peak	p.Arg150Pro (hom)
**FAMILY C**							
Proband (II.3)	21	Female	20/125	Focal schitic changes with predominant subretinal fluid	Normal	Absent light peak	p.Arg47His (het) p.Val216Ile (het)
Sibling (II.1)	11	Male	20/30	Focal schitic changes with predominant subretinal fluid	Not done	Not done	p.Arg47His (het) p.Val216Ile (het)
Sibling (II.4)	26	Male		Focal schitic changes with predominant subretinal fluid	Not done	Not done	p.Arg47His (het) p.Val216Ile (het)
Sibling (II.3)	20	Female		Focal schitic changes with predominant subretinal fluid	Not done	Not done	p.Arg47His (het) p.Val216Ile (het)
**FAMILY D**							
Proband (II.1)	11	Male	20/40	Subretinal fluid with vitelliform deposits	Normal	Absent light peak	p.Thr91Ile (het)
Father (I.1)	38	Male	20/20	Very small sub-foveal lesion	Not done	Not done	p.Thr91Ile (het)
Sibling (II.2)	8	Female	20/20	Normal	Not done	Not done	p.Thr91Ile (het)

^*^hom - homozygous; het - heterozygous.

#### Family A

In family A, two members were affected (Fig. 1a; Table 1). The proband (II.1) was a twenty one year old male with a BCVA of 20/60 in both eyes with hypermetropic refraction. No anterior segment abnormalities were observed in the proband. Fundus image revealed very sparse sub-retinal fibrosis with deposits (Fig. 1b). The affected sibling (II.2) also presented with a normal anterior segment with the corresponding fundus image displaying less conspicuous changes (Fig. 1c). Fundus autofluorescence (FAF) images for both proband and sibling indicated few areas of hyper autoflourescence (Fig. 1d,e). Optical coherence tomography (OCT) images of the proband revealed cystoid and schitic changes in inner and outer nuclear layers of retina (Fig. 1f). Similar OCT features were observed in the affected sibling (Supplementary Fig. S1a). Electrophysiological tests indicated rod cone dysfunction in ERG (Supplementary Fig. S1b–e) with absent light peak in EOG for both proband and the sibling (Supplementary Fig. S1f–i). The electrophysiologic characteristics were similar to the earlier reported cases of ARB^35^. No ocular abnormalities were observed in the unaffected parents. Although the proband and affected sibling were clinically managed with topical and oral carbonic anhydrase inhibitors, the macular fluid persisted.

#### Family B

The proband was a twenty four year old male (from non-consanguineous parents) (Fig. 2a) with a BCVA of 20/30 OD and 20/40 OS. Proband’s fundus exhibited sub-retinal fibrosis with yellowish white, vitelliform deposits located primarily outside the arcade (Fig. 2b) and peripapillary area. These deposits were clearly evident on the FAF image (Fig. 2c). Corresponding OCT images exhibit intra-retinal and sub-retinal fluid collection (Fig. 2d). The proband’s ERG indicated rod-cone dysfunction and the corresponding EOG was abnormal (Supplementary Fig. S2b–d). No ocular abnormalities were observed in unaffected parents and sibling, which is consistent with ARB.

#### Family C

In this family, four members were affected (Fig. 3a) and the pedigree analysis indicated an autosomal recessive inheritance pattern. The proband (II.1) presented with a history of decreased central vision and a BCVA of 20/125 in both eyes. This subject was diagnosed with angle closure glaucoma along with juvenile retinoschisis and had previously undergone peripheral iridotomy. Fundus examination (Fig. 3b) revealed yellowish white sub-retinal precipitates with corresponding hyper autoflourescence on FAF (Fig. 3c) that was primarily concentrated around the posterior pole. Two affected brothers (patient II.1 and II.4) presented with similar clinical features but the yellowish lesions were more confluent, whereas, the affected sister demonstrated less confluent lesions(Fig. 3d–i). OCT analysis (Fig. 3j) of the proband revealed sub-retinal yellowish lesions and scars which corresponded with the hyper-reflective accumulations on or within the RPE cell layer with serous sub-retinal fluid. The OCT features were essentially similar across all the affected members of family C and correlated with the clinical features (Supplementary Fig. S3a–c) exhibiting predominant subretinal fluid with some intraretinal involvement, which was much lower than that observed in families A and B. ERG performed on the proband was normal (Supplementary Fig. S3f,g). However, the corresponding EOG was abnormal with absent light peak (Fig. 3i). The proband underwent multiple surgeries for her glaucoma including bilateral trabeculaectomy and lensectomy. The affected elder brother had glaucoma that was managed medically.

#### Family D

The proband in this family was an eleven year old male (Fig. 4a), who presented with a history of blurred vision and BCVA of 20/40 in both eyes. Fundus examination revealed yellowish, vitelliform deposits in the macula exhibiting a pseudohypopyon appearance (Fig. 4b) that is appreciated better in the FAF image (Fig. 4c). The proband’s father was asymptomatic with visual acuity of 20/20 OU but displaying vitelliform lesion in the left eye (Fig. 4d,e and Supplementary Fig. S4a). The younger sibling of the proband did not present any changes in the retina at the time of examination (Fig. 4f,g and Supplementary Fig. S4b). OCT of the proband indicated sub-retinal deposits with sub-retinal fluid in both eyes (Fig. 4h). The ERG was normal while EOG was abnormal in the proband (Supplementary Fig. S4c–f).

### Exome sequencing and causal variant identification

**Figure 5:**
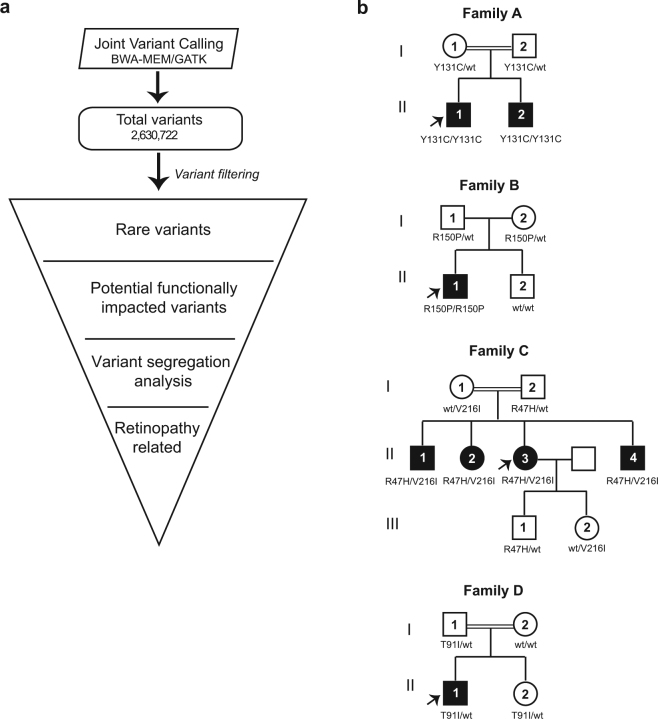
Identification of casual variants in bestrophinopathy families. (**a**) Flowchart depicting the analysis of exome data from bestrophinopathy patients and their relatives. (**b**) Pedigrees of four bestrophinopathy families analyzed in this study. Candidate variants identified in each family is shown. White circles - unaffected females; white squares - unaffected male; filled circles - affected females; filled squares - affected males. Number within circle/square indicates that the sample was sequenced.

We performed exome sequencing on twenty individuals with at least 45x coverage (Supplementary Fig. S5**)** from four unrelated families (Supplementary Fig. S6**)**. Joint-variant calling using data from twenty samples from the four families in our study resulted in 2,630,722 nucleotide variants (Fig. 5a, Methods). Of these, 317,443 (~12%) were rare variants (MAF <= 1%). We predicted 8,788 of the rare variants to have an impact on protein function and/or have disease relevance. The pathogenic variants that segregated through affected and unaffected family members were filtered after overlaying the disease inheritance pattern observed in each pedigree. Additional filters were applied for genes previously implicated in retinopathy as reported on RetNet (https://sph.uth.edu/retnet/). We found *BEST1* mutations in all four families - p.Tyr131Cys in Family A, p.Arg150Pro in Family B, p.Arg47His and p.Val216Ile in Family C, and p.Thr91Ile in Family D. (Fig. 5a,b, Table 1 and Supplementary Table S1).

In Family A, a homozygous transition c.392 A > G in *BEST1* was found in both affected children (proband II.1 and his brother II.2) resulting in a substitution of tyrosine at codon 131 with cysteine (p.Tyr131Cys) (Fig. 5b, Supplementary Fig. S7). Both SIFT^36^ and Polyphen-2^37^ predicted this mutation to be deleterious (Supplementary Table S1). Consistent with the clinical observation, both unaffected parents (I.1 and I.2) were found to be heterozygous for this *BEST1* mutation confirming an autosomal recessive mode of inheritance. Interestingly, in this family OCT showed predominant intraretinal schitic and cystoid changes with negligible subretinal fluid.

In family B, we found a missense mutation, p.Arg150Pro, in the *BEST1* gene that followed a recessive mode of inheritance (Fig. 5b, Supplementary Fig. S8). Consistent with the clinical findings, *BEST1* p.Arg150Pro was observed to be homozygous in the affected proband II.1, heterozygous in both the unaffected parents (I.1 and I.2) and was absent in the unaffected brother (II.2). This alteration was not reported in the 1000 Genome Project^31^ or the ExAC database^33^ and was predicted to be deleterious by both SIFT^36^ and Polyphen-2^37^.

**Figure 6:**
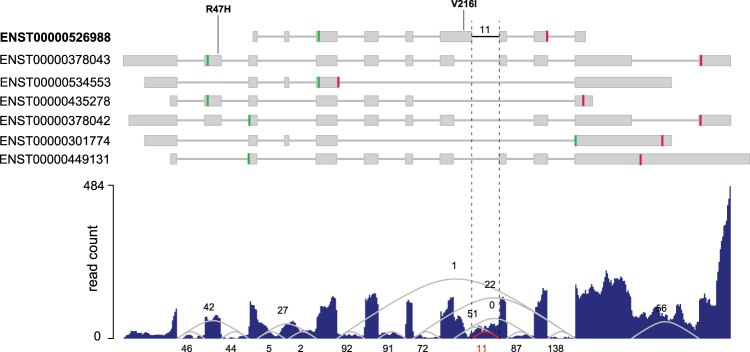
RNA-seq analysis of BEST1 transcript isoforms. Top panel shows structure of seven human protein-coding BEST1 transcript isoforms annotated by Ensembl (release 75), and the location of two mutations p.Arg47His and p.Val216Ile. Gray boxes represent exons. Green vertical line and red vertical line indicate ORF start and stop, respectively. Bottom panel shows average per-base read coverage and junction read counts (middle).

Analysis of family C identified two mutations, p.Arg47His and p.Val216Ile, in the *BEST1* gene that showed a recessive segregation pattern within the family (Fig. 5b, Supplementary Figs S9–10). The p.Arg47His mutation resulted from a transition c.140 G > A in exon 2 of *BEST1* transcript ENST00000378043. However, the p.Val216Ile mutation was a result of a transition c.646 G > A at exon 6 of another *BEST1* transcript ENST00000526988. ENST00000378043 (RefSeq - NM_0041830), encoding a 585 amino acid protein isoform, is the best characterized mRNA transcribed from *BEST1*. ENST00000526988 is a poorly characterized transcript (RefSeq - XM_017018230), encoding a shorter protein isoform (329aa) and its exon 6 is unique among all 7 protein-coding transcripts of *BEST1* (Fig. 6). Manual review of all *BEST1* variants found this compound heterozygous mutation, p.Arg47His and p.Val216Ile, to follow a recessive inheritance mode in this family.

We confirmed the expression of both ENST00000378043 and ENST00000526988 isoforms in retina by analyzing RNA-seq data obtained from the retinal tissue of a healthy donor (see Materials & Methods). RNA-seq split-reads (n = 11) supporting the intron junction between exon 6 and exon 7 of ENST00000526988 confirmed that this alternate transcript is expressed in the retina and hence likely to be a relevant isoform of *BEST1* in the eye (Fig. 6).

All four affected members in family C (including proband) carried both the p.Arg47His and p.Val216Ile mutations. The unaffected members (parents and children of proband) were carriers for either p.Arg47His or p.Val216Ile. The heterozygous p.Arg47His mutation was reported in a German patient with adult-onset vitelliform macular dystrophy (AVMD)^38^ and, more recently, in a Chinese patient with Best Vitelliform Macular Dystrophy (BVMD)^39^. The p.Val216Ile mutation was not present in the 1000 Genome Project^31^ or the ExAC database^33^ and was predicted to be damaging by SIFT^36^ and benign by Polyphen-2^37^.

In family D, we did not observe any homozygous pathogenic mutations through the segregation analysis. However, upon manual review, the proband (II.1) was found to have a heterozygous p.Thr91Ile mutation in the *BEST1* gene which was predicted to be deleterious by both SIFT^36^ and Polyphen-2^37^ (Fig. 5b, Supplementary Fig. S11, Supplementary Table S1). The same mutation was also observed in the father and sister but was absent in the mother. The father (I.1) who did not have any clinical symptoms, demonstrated vitelliform lesions in his left eye, indicating that the mutation may show an incomplete penetrance.

## Discussion

The *BEST1* gene has been associated with a wide range of ocular phenotypes. Although clinical presentation of the autosomal recessive form of bestrophinopathy is distinct from the autosomal dominant form, both these conditions are known to arise from *BEST1* (*VMD2*) gene alterations. Mutations in *BEST1* gene have pleiotropic effects and the phenotypes observed are influenced by age, gender, environment, epigenetic factors and presence of modifier genes^7,40^.

Our study included four families where probands from three were classified as ARB (families A, B and C) and the proband from the fourth (Family D) was suspected to have BVMD (family D) based on clinical observations. The *BEST1* mutations and the pattern of inheritance confirmed the ARB clinical diagnosis in family A and B. Interestingly, the sequencing results established the family C proband to carry a compound heterozygous *BEST1* mutation that likely led to ARB. Finally, in family D we identified a potential autosomal dominant *BEST1* mutation that exhibited an incomplete penetrance leading to BVMD in the affected proband.

**Figure 7:**
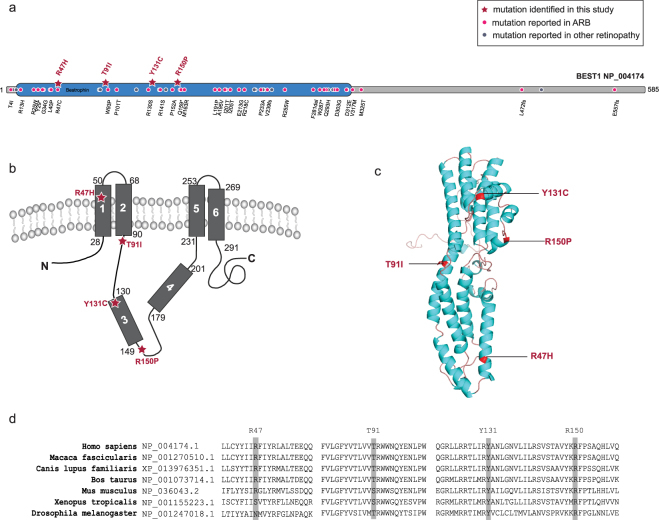
BEST1 mutations depicted on (**a**) a cartoon of BEST1 with the conserved pfam domain, (**b**) the predicted topology of BEST1^56^, (**c**) the structure of BEST1 (ProteinModelPortal, ID O76090), and (**d**) a multiple sequence alignment of BEST1 protein sequences from multiple species. ARB - Autosomal Recessive Bestrophinopathy.

In Family A, we observed a homozygous p.Tyr131Cys *BEST1* mutation. The Y131 is a conserved residue (Fig. 7d) and substitution at this position likely affects BEST1 function. The p.Tyr131Cys mutation has not been previously reported. Further, in family B we found a novel homozygous c.449 G > C transversion that led to a replacement of a highly conserved arginine to proline at codon 150. Family A and Family B correlated both genotypically and phenotypically to ARB.

In family C, the flecks on fundus imaging and autofluorescence tests showed widespread posterior pole distribution with very minimal but definitive focal schitic changes with predominant subretinal fluid, suggestive of ARB. However, phenotypically, the affected members of family C were dissimilar to those of families A and B. In particular, the intraretinal involvement observed in OCT images from family C was relatively lesser compared to families A and B. Interestingly, in family C we observed two missense substitutions that map to two different isoforms of *BEST1*. One mutation led to arginine to histidine change at codon 47 (p.Arg47His; ENST00000378043) while the other mutation was a valine to isoleucine substitution at codon 216 (p.Val216Ile; ENST00000526988) of a previously known alternate *BEST1* transcript. We confirmed the expression of this alternate transcript in normal donor retinal tissue by RNA sequencing. The p.Arg47His mutation was identified previously in a BVMD^39^ and an AVMD case^38^ although, the inheritance patterns were not reported. BVMD and AVMD caused by *BEST1* mutations are known to show incomplete inheritance and penetrance^41^. A recent study functionally assessed the p.Arg47His mutation and suggested that it might be a mildly impaired mutant^42^. Given these, it is possible that in the unaffected carriers of family C, the disease was not penetrant in the absence of the additional BEST1 mutation observed in the proband and other affected family members.

A *BEST1* compound heterozygous mutation, p.Trp29X/p.Arg141His, was previously described in a Swedish ARB family^43^. Similarly, in another ARB family the affected members were reported to carry heterozygous p.Ala243Glu/p.Arg200X *BEST1* mutations^44^. To the best of our knowledge, our study is the first report of a compound heterozygous mutations involving two *BEST1* isoforms. However, the molecular function of the alternate transcript ENST00000526988 and the impact of compound heterozygous mutations (p.Arg47His and p.Val216Ile) on the function of *BEST1* in associated disease phenotypes require additional studies.

Our analysis of family D found a heterozygous *BEST1* p.Thr91Ile mutation in the affected proband. Remarkably, the proband’s father also carried this mutation but was only mildly affected with no loss of vision; only a small foveal lesion was observed by OCT and FAF. Incomplete penetrance in BVMD has been reported^45^ and it should be noted that variable presentation of the disease as well as lack of clinical symptoms are also associated with mutations in certain families^46^. While the *BEST1* p.Thr91Ile mutation identified in family D is suggestive of a dominant mutation, the mild phenotype in the father is indicative of the variable penetrance of this mutation that might be modulated by other background genomic variations. Consistent with this, incomplete penetrance of an autosomal dominant *BEST1* mutation has been reported in multiple studies^35,47,48^. Interestingly, in 23 patients with heterozygous *BEST1* mutations, six were found to have no clinically detectable disease related phenotype^49^. While the effect of *BEST1* mutations on its function remains to be fully elucidated, a dominant negative role for some mutations^50,51^ and a functional role for compound heterozygous mutations in *BEST1* disease phenotypes has been proposed based on *in vitro* functional studies^15,52^.

The 585 amino acid long BEST1 protein is characterized by a highly conserved N-terminal region followed by four to six transmembrane domains. BEST1 functions as a calcium-activated chloride channel (CaCC) which regulates the flow of chloride and other monovalent anions across cellular membranes in response to intracellular calcium levels^53^. The N-terminus of this protein (amino acids 1–390) contains all of the putative membrane-spanning domains and is sufficient for its CaCC activity while the carboxy-terminal region (amino acids 391–585 of BEST1) is predicted to be unstructured^54^. Structural models of BEST1 have been proposed, which describe topological positions of the N-terminal region, the transmembrane domains and the C-terminus^55,56^. Such models propose the N- and C-termini as being cytosolic with the presence of four transmembrane domains (domain 1,2 and 5, 6) while domain 3 and 4 are cytoplasmic^55,56^ (Fig. 7a–c). The crystal structure of chicken BEST1, which is 74% identical to human BEST1, has been solved. This structure is distinct from other channel proteins and is comprised of a pentamer of five BEST1 subunits symmetrically arranged around a central axis which creates a pore for chloride ions to pass through the protein complex^57^. Although the detailed mechanisms that lead to the disease are not fully understood, most of the characterized *BEST1* mutations alter electrophysiological properties of the channel^51,54,57^. Crystallographic studies of wild-type and mutant proteins suggest that *BEST1* mutations altering the cytoplasmic pore structure affects the permeability of anions or anion-cation selectivity^58^. Interestingly, the mutations discovered in this study are localized to the N-terminal region (Fig. 7a–c). The mutations identified in family A (p.Tyr131Cys) and family B (p.Arg150Pro) lie in the third cytoplasmic domain. Mutations observed in family C (p.Arg47His) localize to the first transmembrane domain, while the mutation in family D (p.Thr91Ile) alters an amino acid proximal to the second transmembrane domain. The amino acids at these positions are conserved among mammals (Fig. 7d).

## Electronic supplementary material

Supplementary Figures S1-S11

Supplementary Table S1
