# Supplementary material for: Next generation sequencing identifies novel disease-associated BEST1 mutations in Bestrophinopathy patients
Source: Sci Rep. 2018 Jul 5;8:10176. doi: 10.1038/s41598-018-27951-8 (PMC6033935; doi:10.1038/s41598-018-27951-8)

Supplementary figures:

Next generation sequencing identifies novel disease-associated BEST1 mutations in Bestrophinopathy patients

Thong T Nguyen[2#], Poornachandra B[4#], Anshuman Verma[1], Ruchir A Mehta[4], Sameer Phalke[3], Rajani Battu[4], Vedam L Ramprasad[3], Andrew S. Peterson[2*], Arkasubhra Ghosh[1*], Somasekar Seshagiri[2*]
[1]GROW Research Laboratory, Narayana Nethralaya Foundation, Bangalore, IN.
[2]Department of Molecular Biology, Genentech Inc, USA.
[3]Medgenome Inc., Bangalore, IN
[4]Retina Department, Narayana Nethralaya, Bangalore, IN
[#] These authors contributed equally to the study.

Short title: Mutations in Best disease
Key Words: Retina, Bestrophin, Vitelliform macular degeneration, exome sequencing.

***Corresponding authors:**
Andrew Peterson, PhD
Department of Molecular Biology, Genentech Inc.,
1 DNA Way, South San Francisco, California 94080, USA.
Email: peterson.andrew@gene.com

Arkasubhra Ghosh, MSc, PhD
GROW Research Laboratory
Narayana Nethralaya Foundation, Narayana Health City,
258/A, Bommasandra, Hosur Road, Bangalore - 560 099 - INDIA.
Email: arkasubhra@narayananethralaya.com

Sekar Seshagiri, PhD
Department of Molecular Biology, Genentech Inc.,
1 DNA Way, South San Francisco, California 94080, USA.
Email: sekar@gene.com

Supplementary Figure Legends:

Supplementary Figure S1: FAMILY A. Sibling (II.2, a) OCT of the sibling showing cystoid (white Asterix) and schitic changes in inner and outer nuclear layers of retina. ERG of the proband (b, c) and sibling (II.2, d, e) showing rod cone dysfunction. EOG of the proband (f, g) and sibling (II.2, h, i) showing minimal light rise and no light rise, respectively.

Supplementary Figure S2: FAMILY B. (a, b) ERG showing rod cone dysfunction in proband. EOG (c, d) showed an absence of light peak in the proband.

Supplementary Figure S3: Family C. (a-c) OCT showing hyper reflective RPE cell Layer(Black Arrow) with a serous subretinal fluid (White Asterix) with focal schitic changes (White Arrow) in proband's affected brother (II.1, a), brother (II.4, b) and sister (II.2, c). (d-g) Proband's (II.3) ERG (d,e) was normal and EOG (f, g) showed absence of light rise.

Supplementary Figure S4: Family D. (a-b) OCT showing focal subretinal fluid (White Arrow) in the left eye of the proband's father (I.1, a) in family D. Sibling (II.2, b) OCT was normal. (c-f) Proband (II.1) ERG (c, d) and EOG (e, f) was abnormal.

Supplementary Figure S5: Exome sequencing depth and coverage across samples sequenced in the study.

Supplementary Figure S6: Kinship coefficients between the sequenced family members confirmed their relationship.

Supplementary Figure S7: Representative Sanger sequence chromatograms of members in family A showing BEST1 p.Tyr131Cys mutation.

Supplementary Figure S8: Representative Sanger sequence chromatograms of members in family B showing BEST1 p.Arg150Pro mutation.

Supplementary Figure S9: Representative Sanger sequence chromatograms of members in family C showing BEST1 p.Arg47His mutation.

Supplementary Figure S10: IGV visualization of reads spanning BEST1 p.Val216Ile mutation in members of family C.

Supplementary Figure S11: Representative Sanger sequence chromatograms of members in family D showing BEST1 p.Thr91Ile mutation.

Supplementary Figure S1

S1a

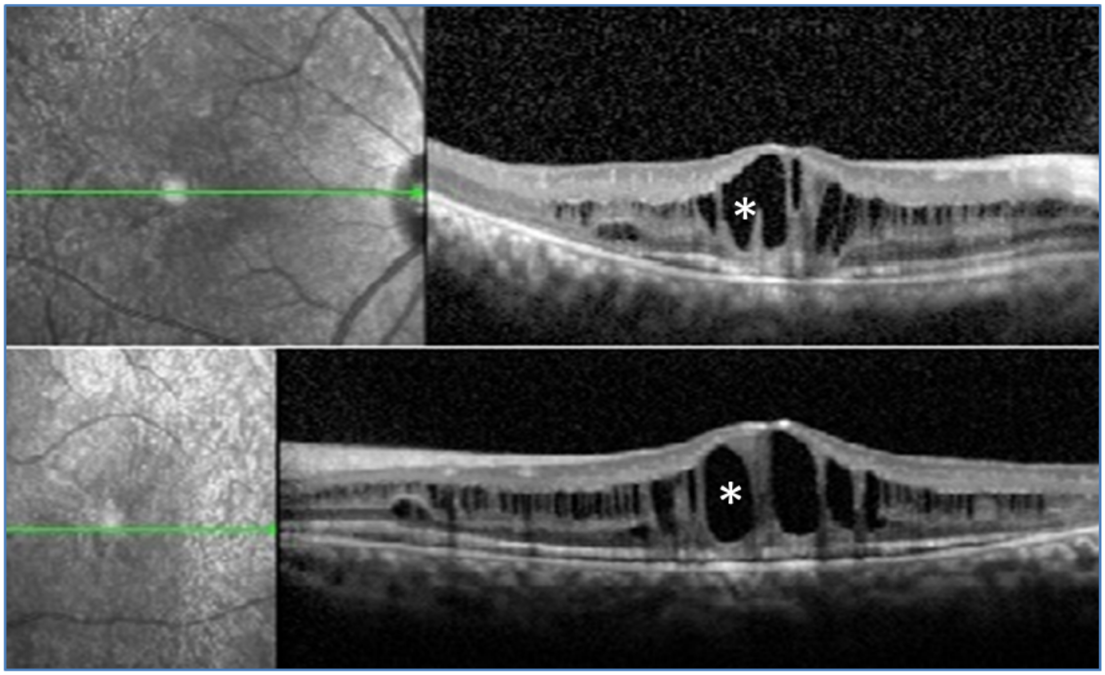

S1b

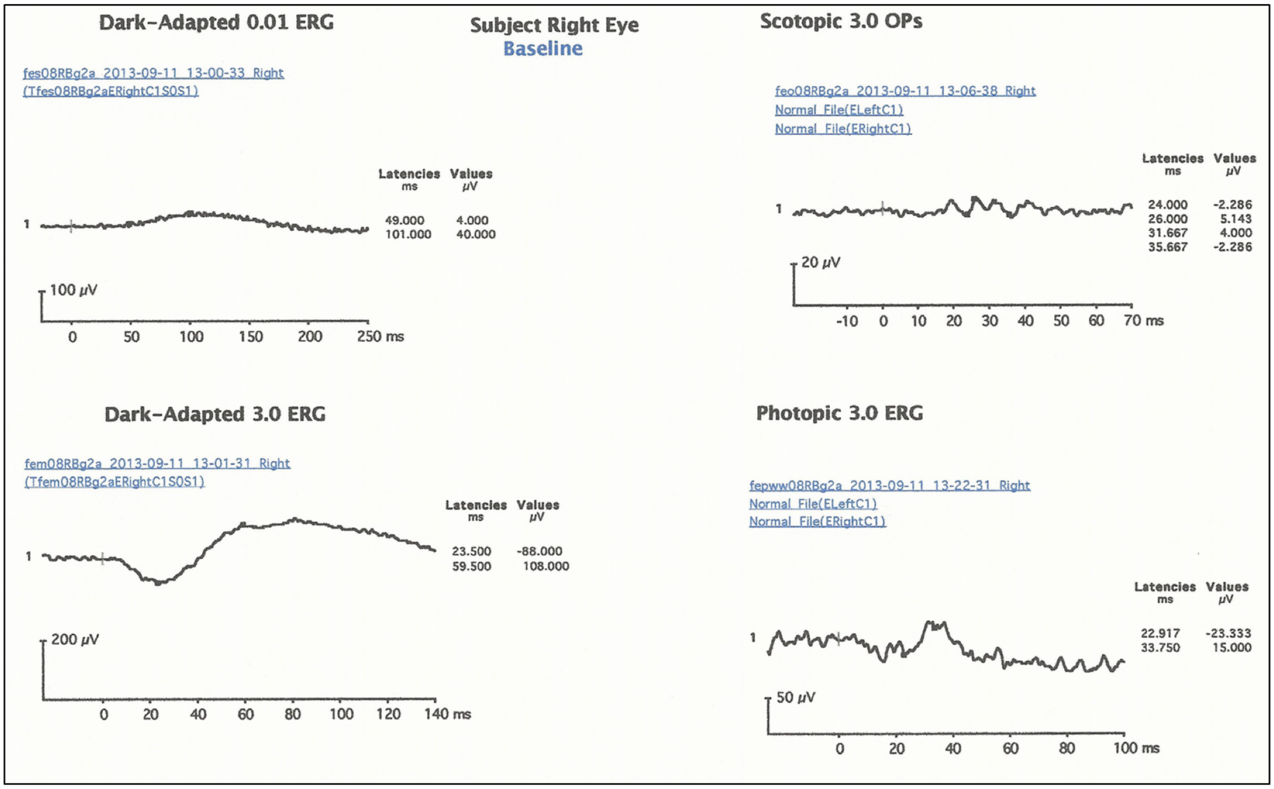

S1c

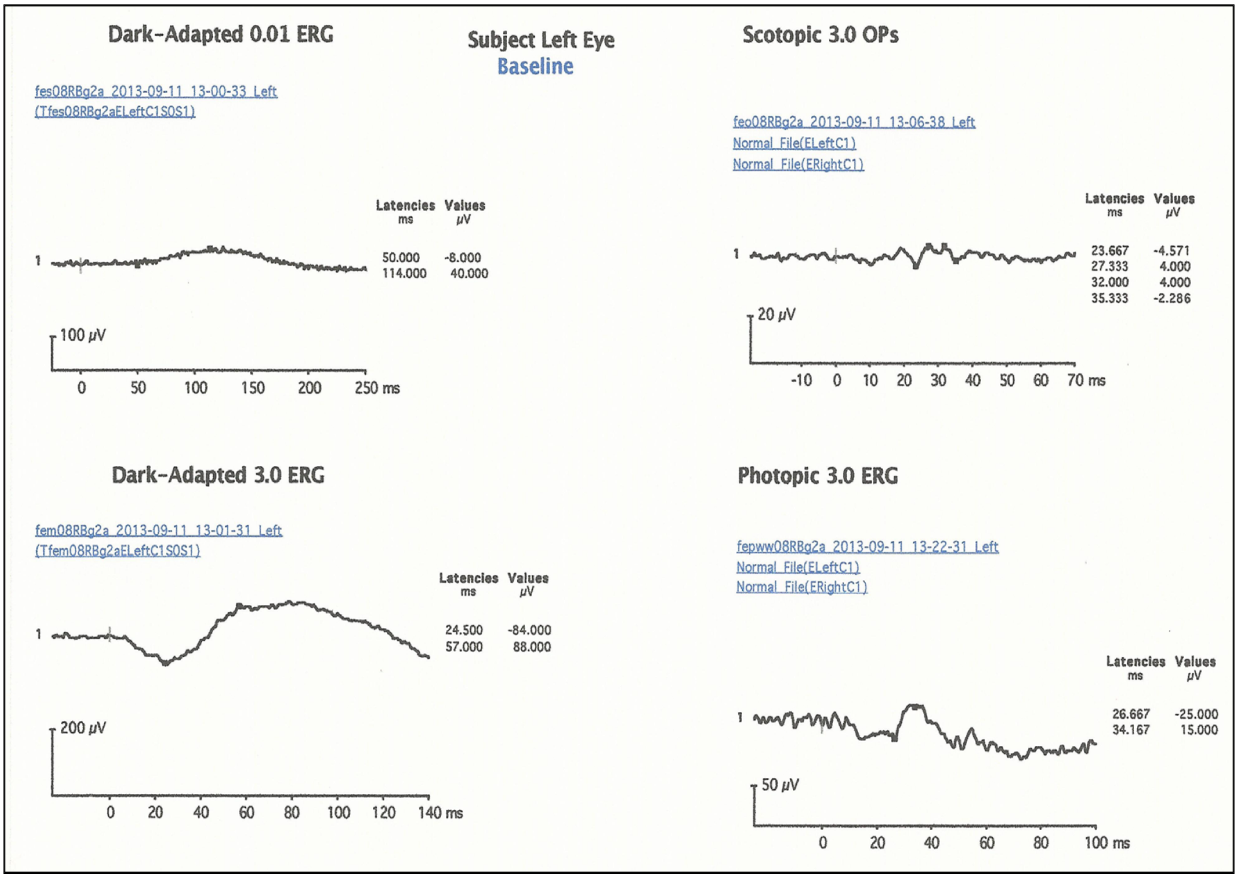
Dark-Adapted 0.01 ERG
Subject Left Eye
Baseline
Scotopic 3.0 OPs
fes08RBg2a 2013-09-11 13-00-33 Left
(Tfes08RBg2aELeftC1S0S1)
Latencies ms Values µV
50.000 -8.000
114.000 40.000
100 µV
0 50 100 150 200 250 ms
feo08RBg2a 2013-09-11 13-06-38 Left
Normal File(ELeftC1)
Normal File(ERightC1)
Latencies ms Values µV
23.667 -4.571
27.333 4.000
32.000 4.000
35.333 -2.286
20 µV
-10 0 10 20 30 40 50 60 70 ms
Dark-Adapted 3.0 ERG
fem08RBg2a 2013-09-11 13-01-31 Left
(Tfem08RBg2aELeftC1S0S1)
Latencies ms Values µV
24.500 -84.000
57.000 88.000
200 µV
0 20 40 60 80 100 120 140 ms
Photopic 3.0 ERG
fepww08RBg2a 2013-09-11 13-22-31 Left
Normal File(ELeftC1)
Normal File(ERightC1)
Latencies ms Values µV
26.667 -25.000
34.167 15.000
50 µV
0 20 40 60 80 100 ms

S1d

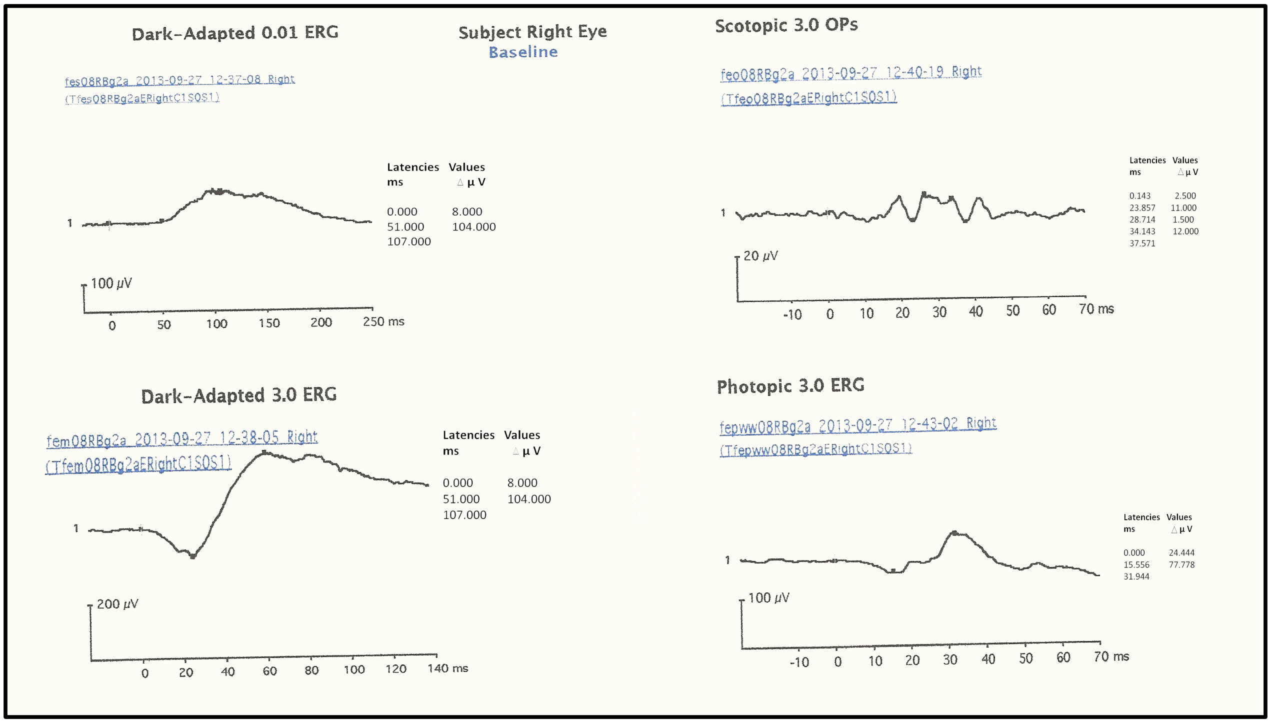
Dark-Adapted 0.01 ERG
Subject Right Eye
Baseline
Scotopic 3.0 OPs
fes08RBg2a 2013-09-27 12-37-08 Right
(Tfes08RBg2aERightC1S0S1)
Latencies ms Values △µV
0.000 8.000
51.000 104.000
107.000
100 µV
0 50 100 150 200 250 ms
feo08RBg2a 2013-09-27 12-40-19 Right
(Tfeo08RBg2aERightC1S0S1)
Latencies ms Values △µV
0.143 2.500
23.857 11.000
28.714 1.500
34.143 12.000
37.571
20 µV
-10 0 10 20 30 40 50 60 70 ms
Dark-Adapted 3.0 ERG
fem08RBg2a 2013-09-27 12-38-05 Right
(Tfem08RBg2aERightC1S0S1)
Latencies ms Values △µV
0.000 8.000
51.000 104.000
107.000
200 µV
0 20 40 60 80 100 120 140 ms
Photopic 3.0 ERG
fepww08RBg2a 2013-09-27 12-43-02 Right
(Tfepww08RBg2aERightC1S0S1)
Latencies ms Values △µV
0.000 24.444
15.556 77.778
31.944
100 µV
-10 0 10 20 30 40 50 60 70 ms

S1e

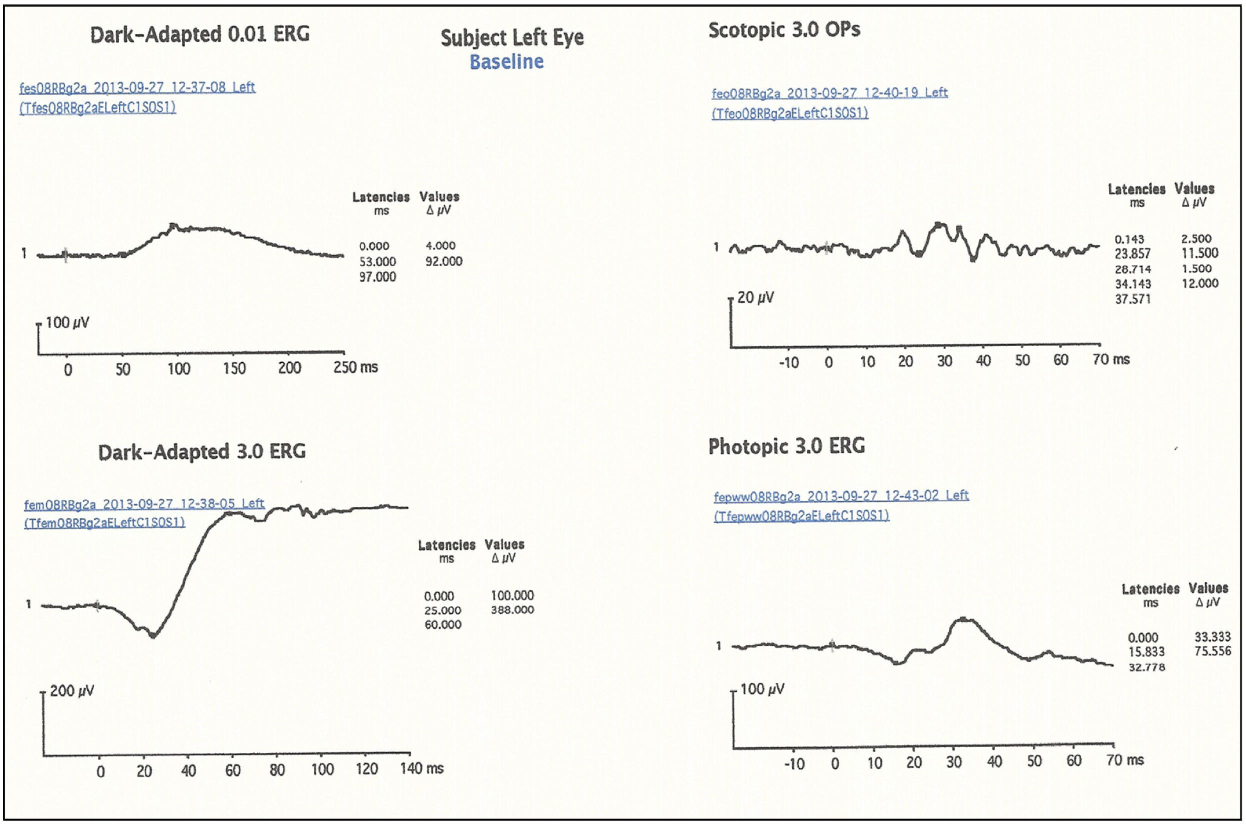

S1f

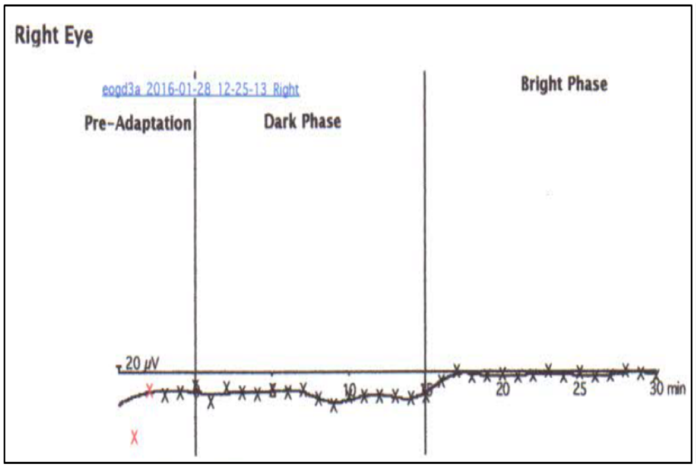

S1g

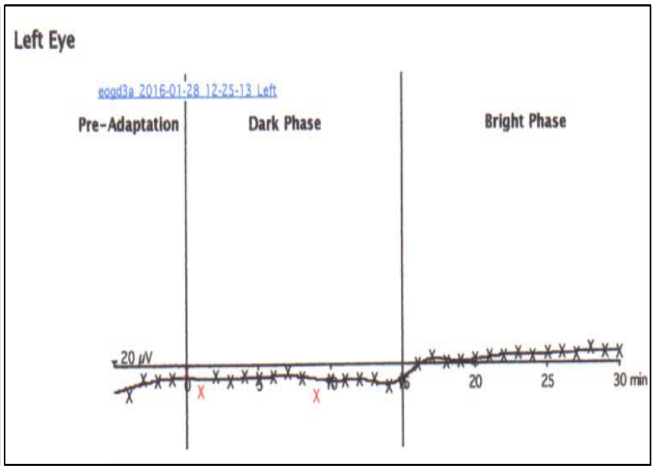

S1h

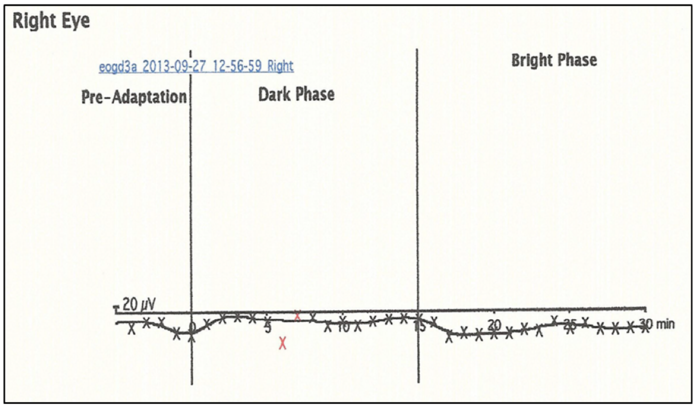

S1i

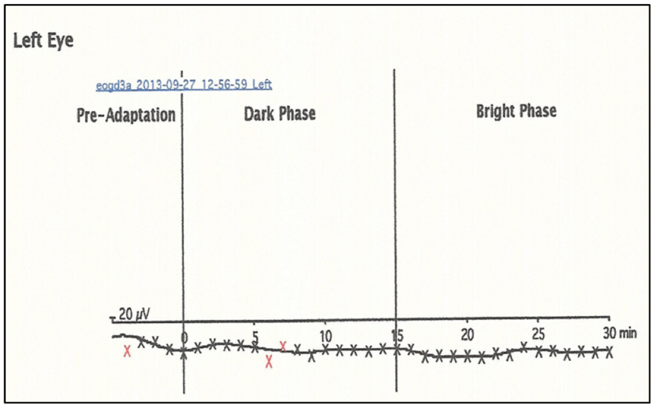

Supplementary Figure S2

S2a

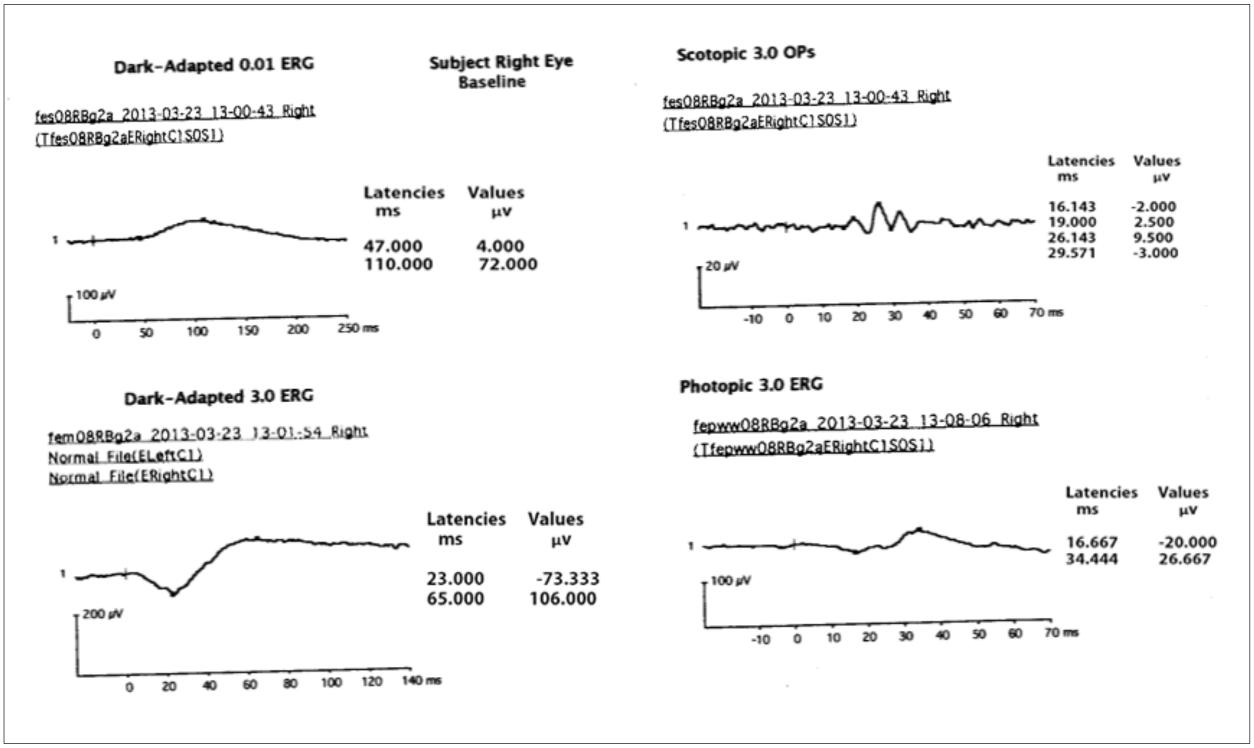

S2b

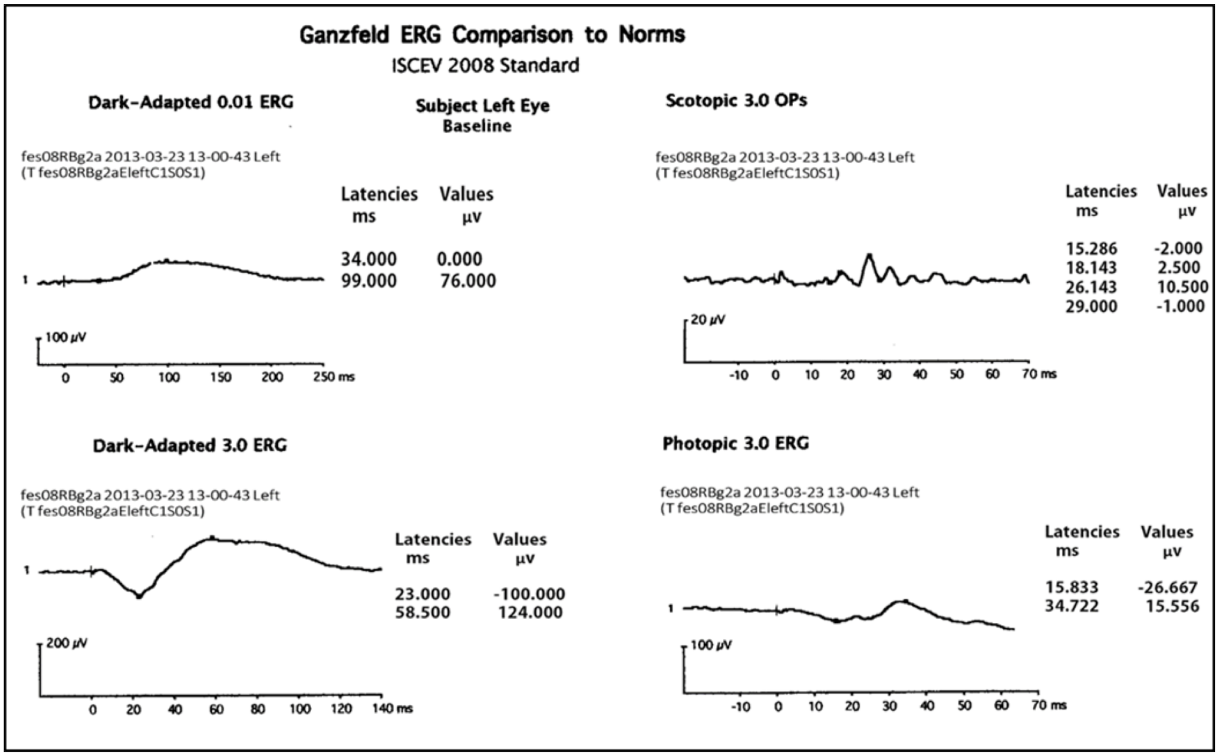

S2c

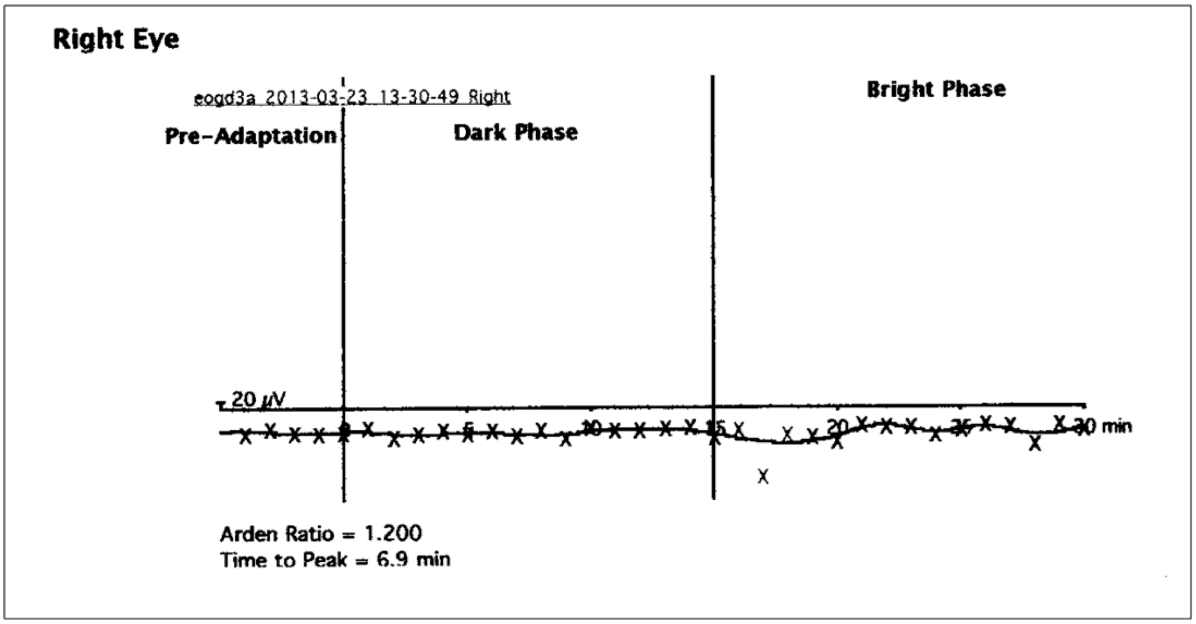
Right Eye
eogd3a 2013-03-23 13-30-49 Right
Pre-Adaptation
Dark Phase
Bright Phase
20 µV
0
5
10
15
20
25
30 min
Arden Ratio = 1.200
Time to Peak = 6.9 min

S2d

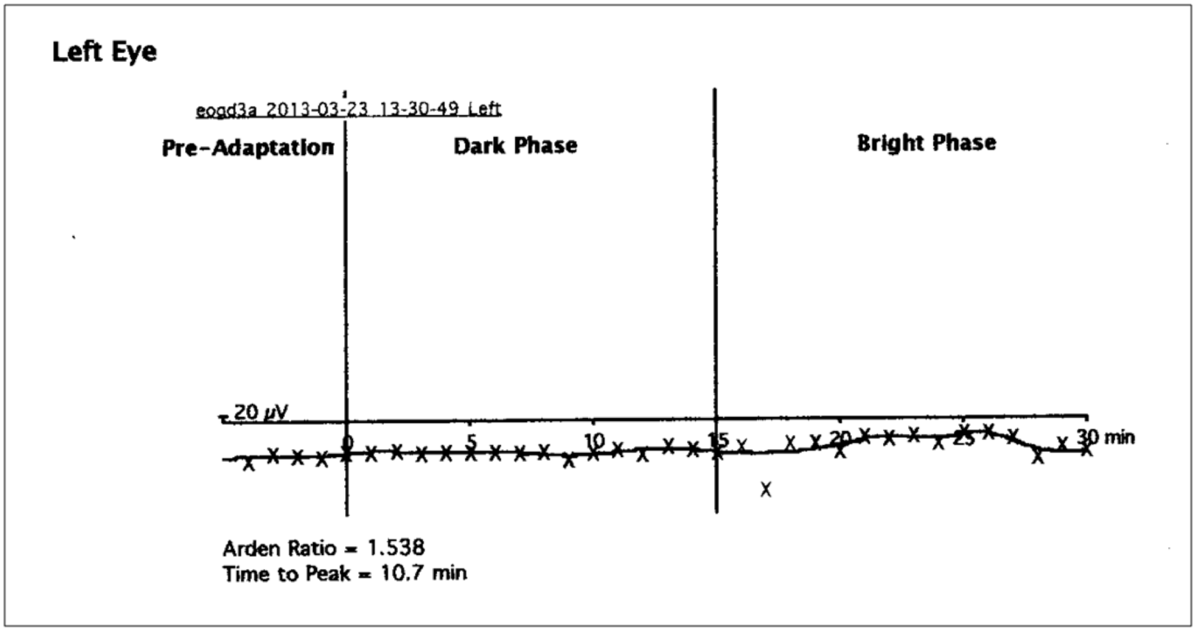
Left Eye
eogd3a 2013-03-23 13-30-49 Left
Pre-Adaptation
Dark Phase
Bright Phase
20 µV
0
5
10
15
20
25
30 min
Arden Ratio = 1.538
Time to Peak = 10.7 min

Supplementary Figure S3

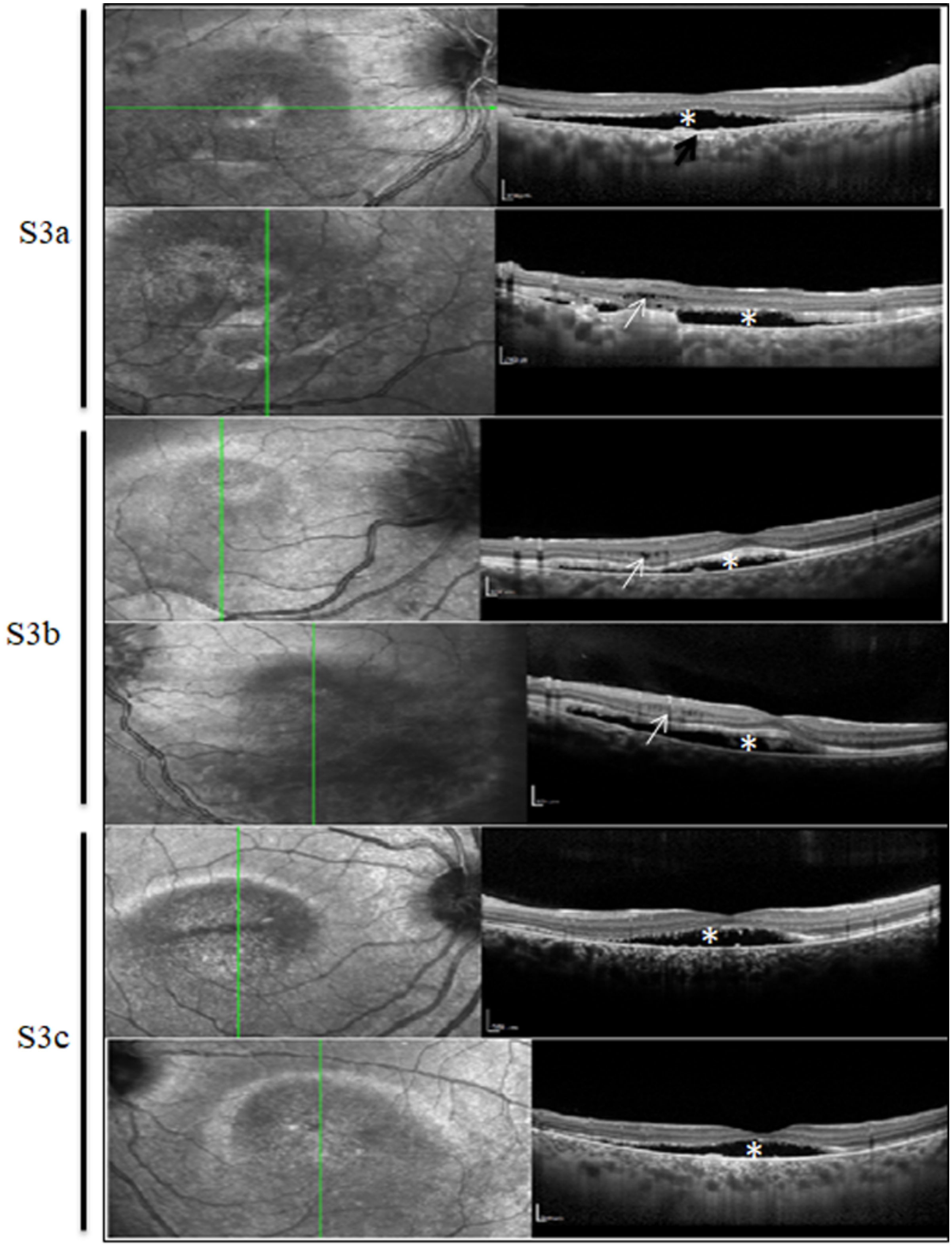

S3d

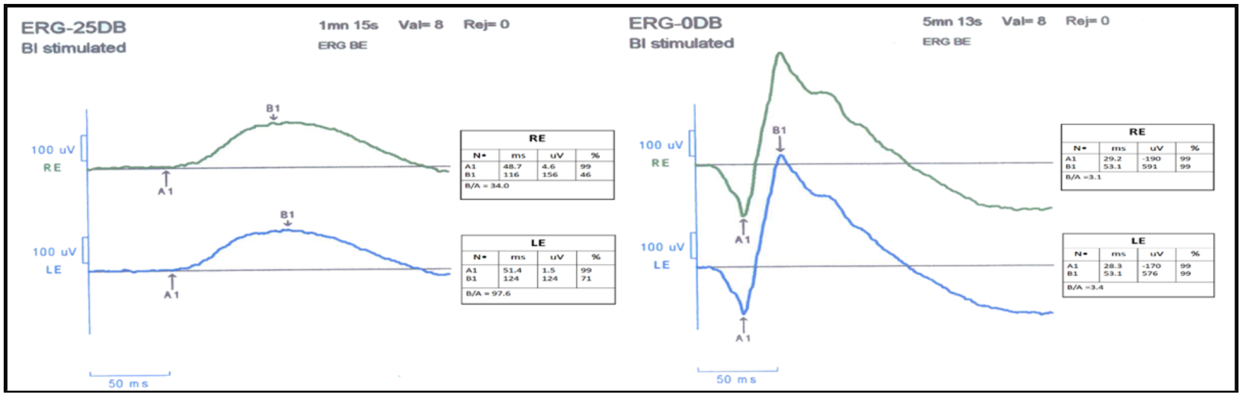

S3e

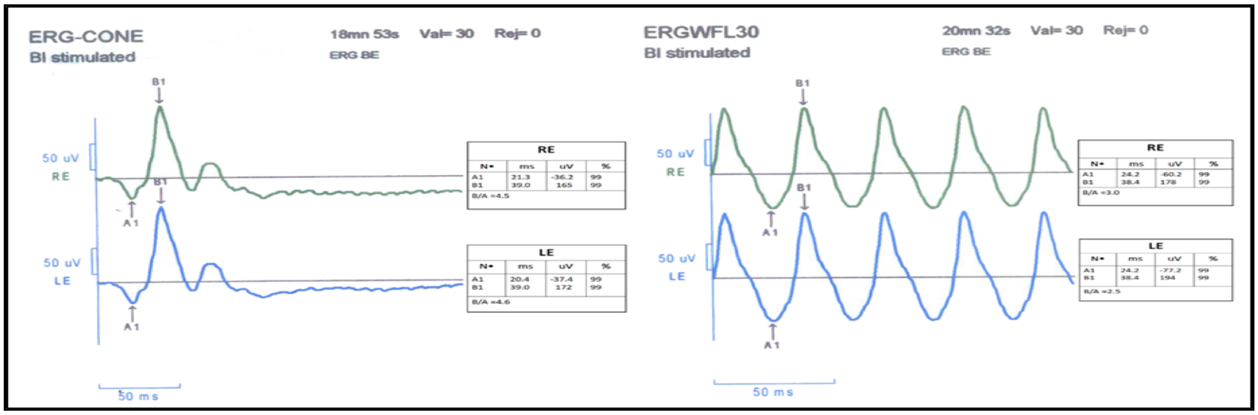

S3f

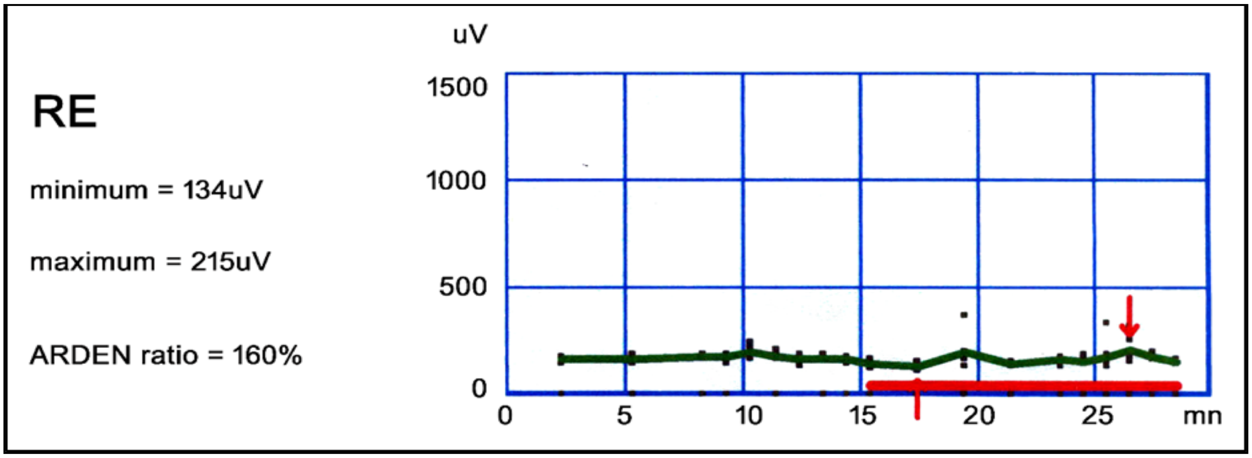

S3g

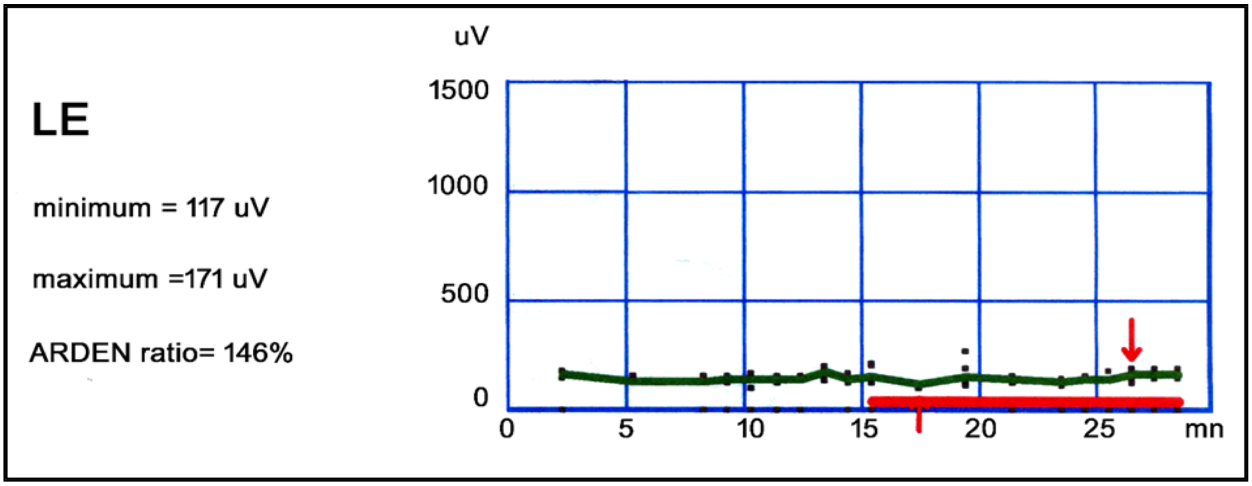

Supplementary Figure S4

S4a

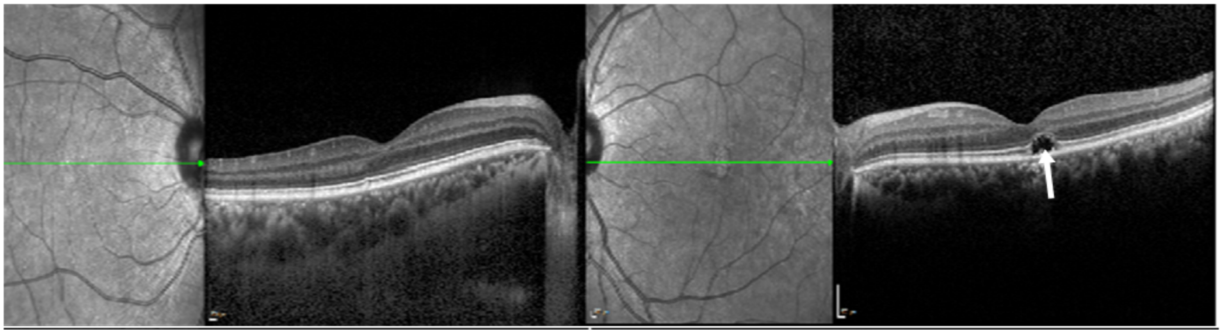

S4b

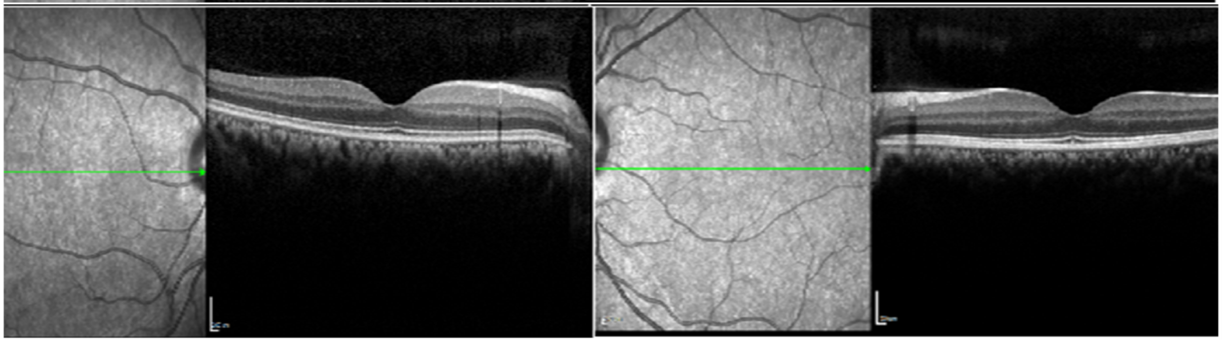

S4c

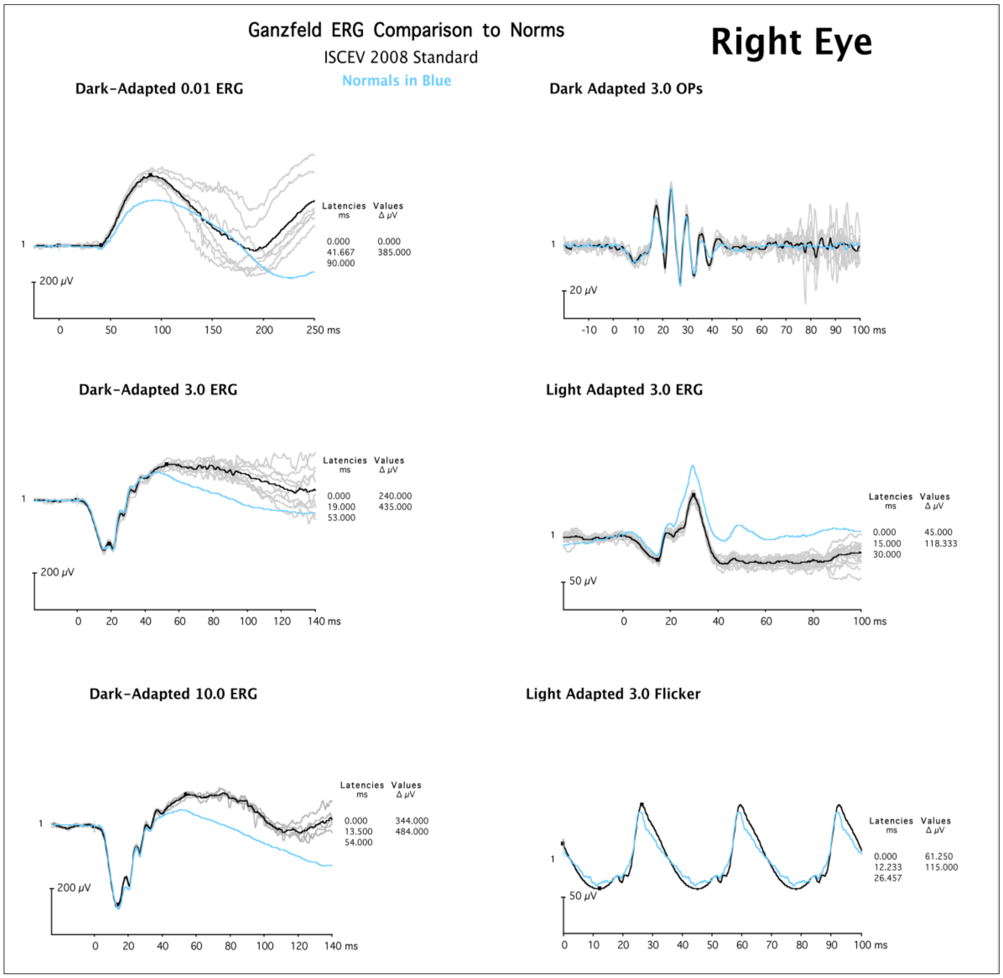

S4d

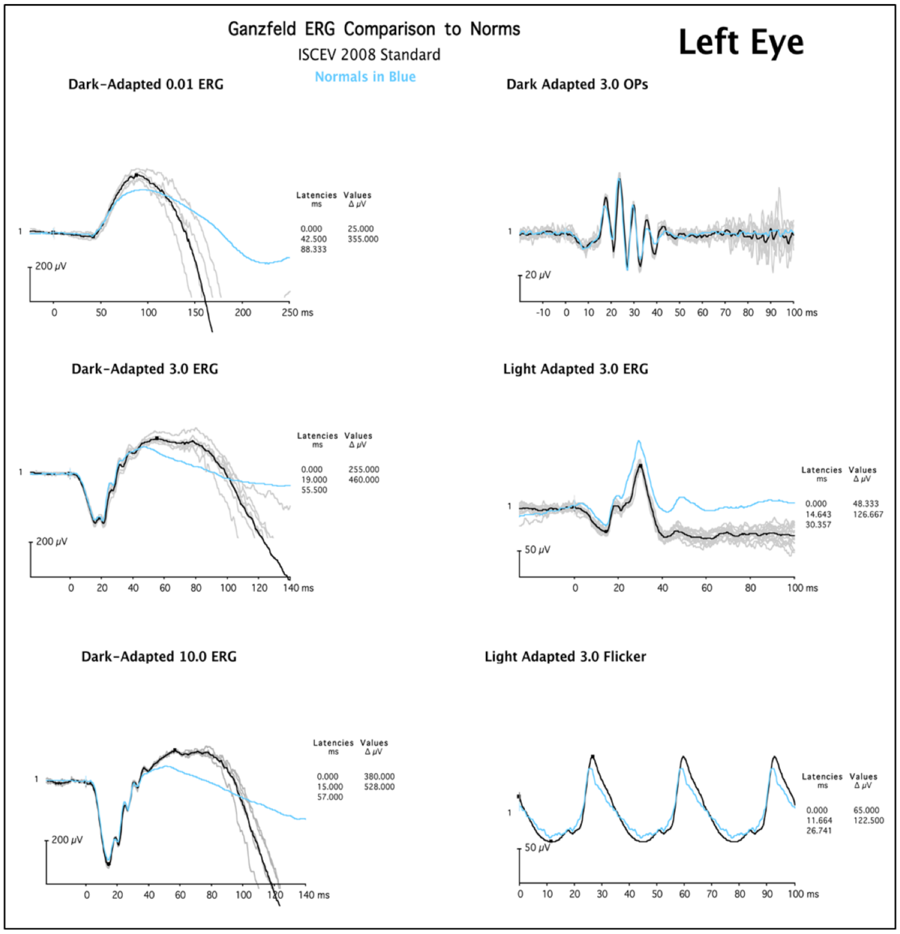
Ganzfeld ERG Comparison to Norms
ISCEV 2008 Standard
Normals in Blue
Left Eye
Dark-Adapted 0.01 ERG
Latencies ms Values Δ µV
0.000 42.500 88.333
25.000 355.000
200 µV
0 50 100 150 200 250 ms
Dark Adapted 3.0 OPs
20 µV
-10 0 10 20 30 40 50 60 70 80 90 100 ms
Dark-Adapted 3.0 ERG
Latencies ms Values Δ µV
0.000 19.000 55.500
255.000 460.000
200 µV
0 20 40 60 80 100 120 140 ms
Light Adapted 3.0 ERG
Latencies ms Values Δ µV
0.000 14.643 30.357
48.333 126.667
50 µV
0 20 40 60 80 100 ms
Dark-Adapted 10.0 ERG
Latencies ms Values Δ µV
0.000 15.000 57.000
380.000 528.000
200 µV
0 20 40 60 80 100 120 140 ms
Light Adapted 3.0 Flicker
Latencies ms Values Δ µV
0.000 11.664 26.741
65.000 122.500
50 µV
0 10 20 30 40 50 60 70 80 90 100 ms

S4e

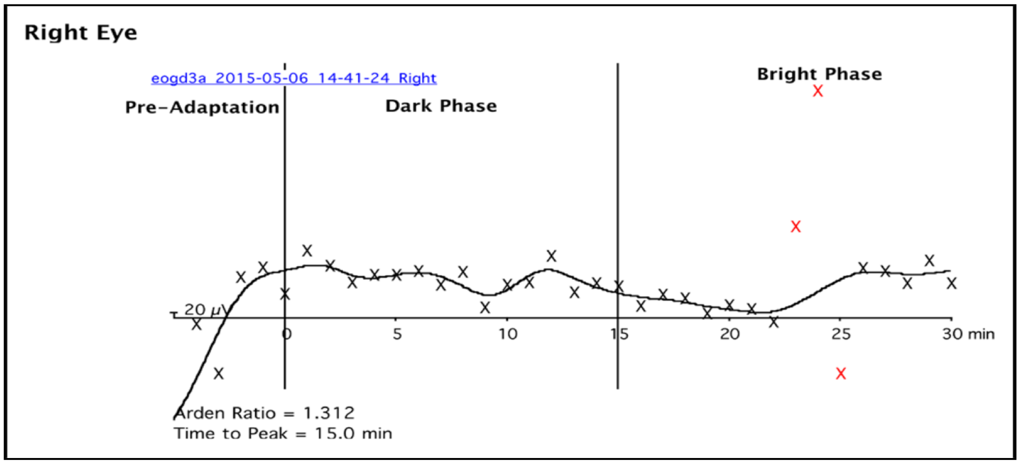
Right Eye
eogd3a 2015-05-06 14-41-24 Right
Pre-Adaptation
Dark Phase
Bright Phase
20 µV
0 5 10 15 20 25 30 min
Arden Ratio = 1.312
Time to Peak = 15.0 min

S4f

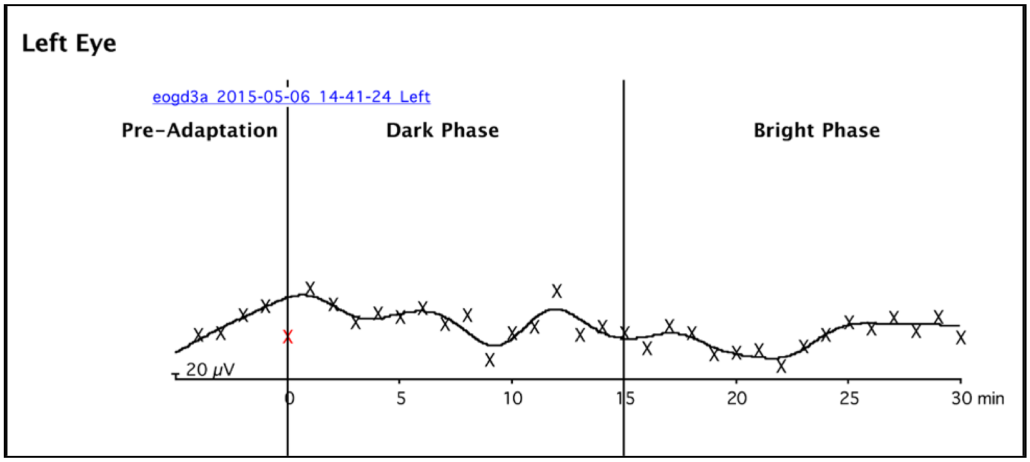
Left Eye
eogd3a 2015-05-06 14-41-24 Left
Pre-Adaptation
Dark Phase
Bright Phase
20 µV
0 5 10 15 20 25 30 min

Supplementary Figure S5

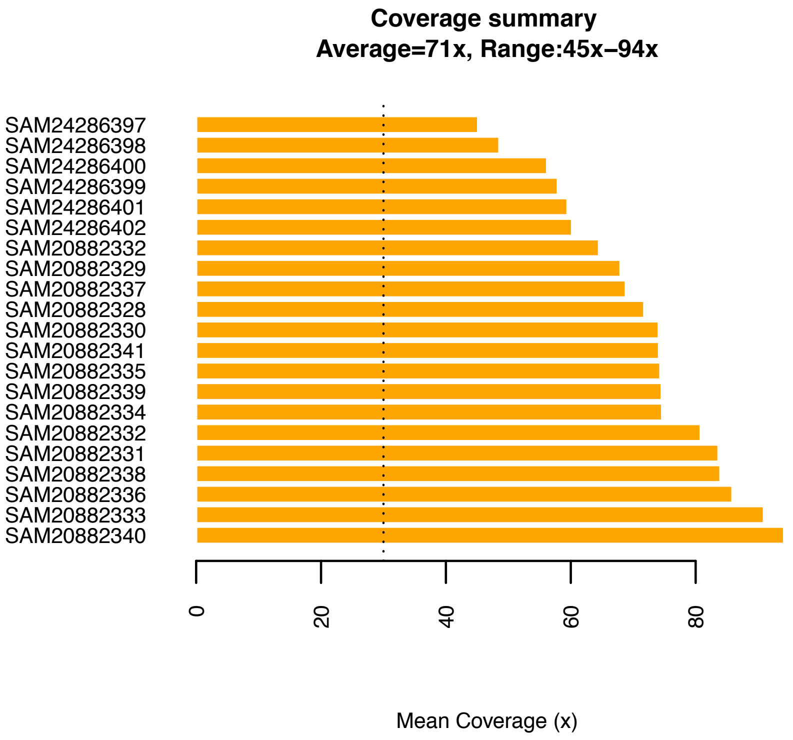

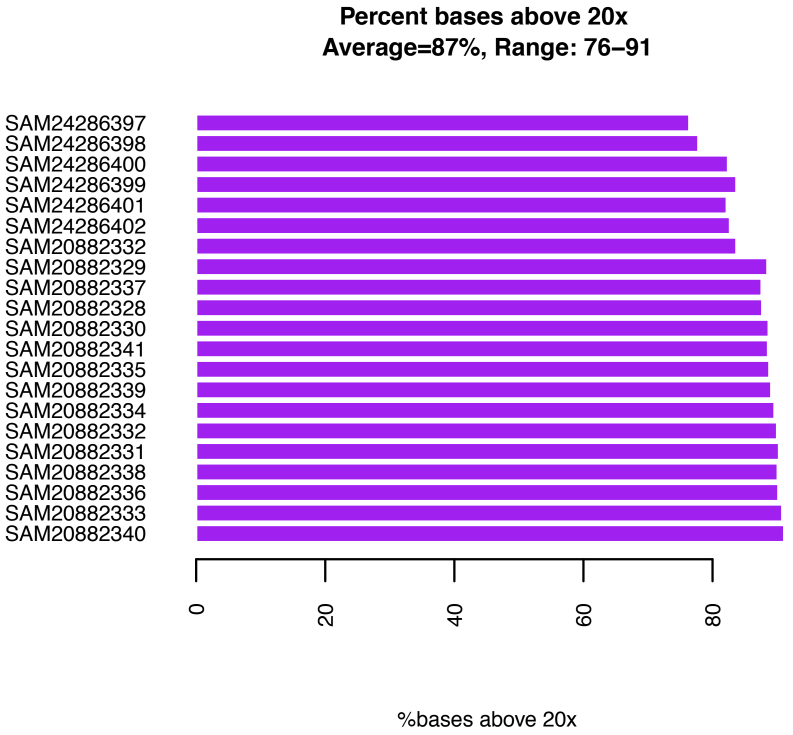

Supplementary Figure S6

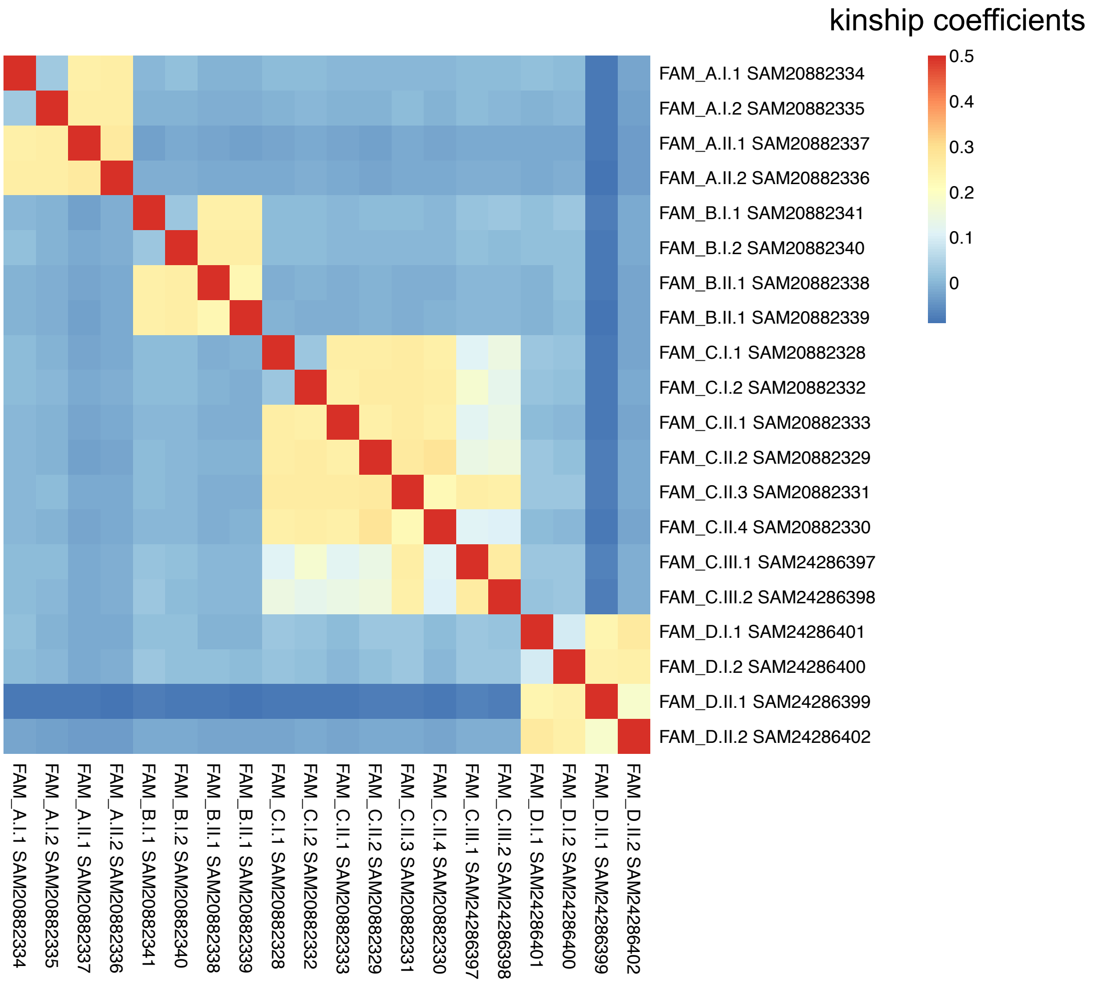

Supplementary Figure S7

Family A

GRCh37 Chr11; 61723334 A>G; p.Y131C

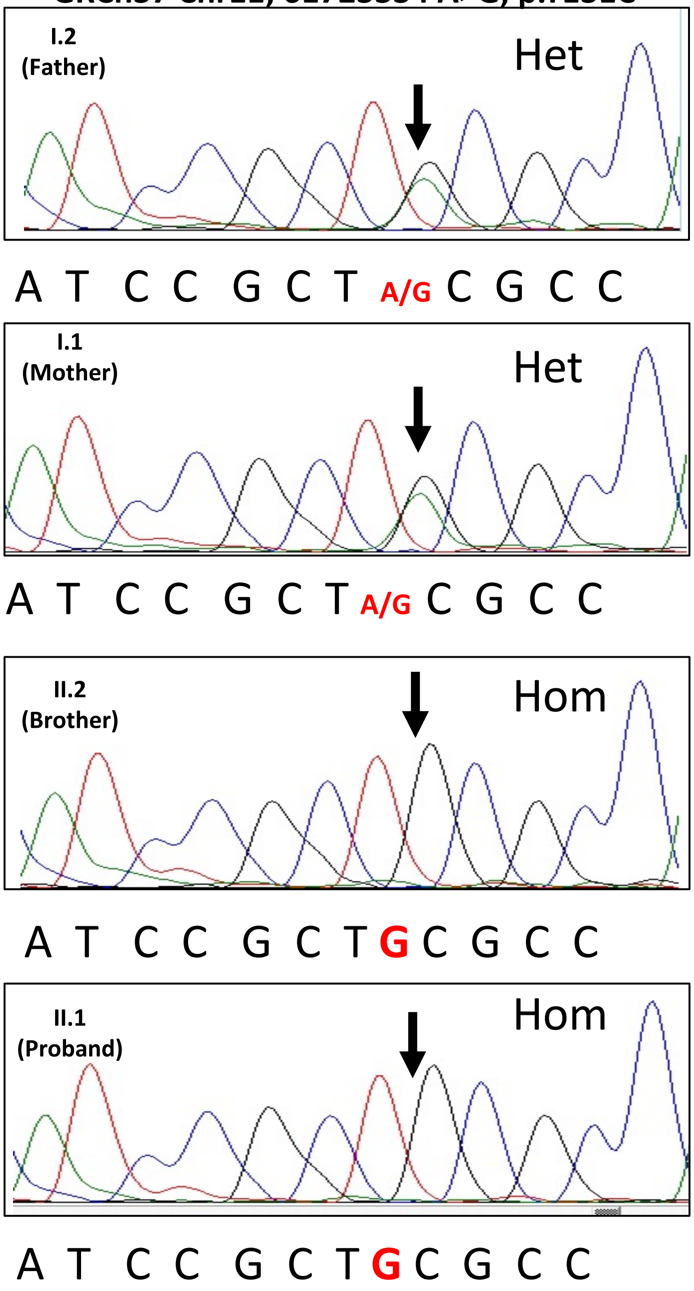

Supplementary Figure S8

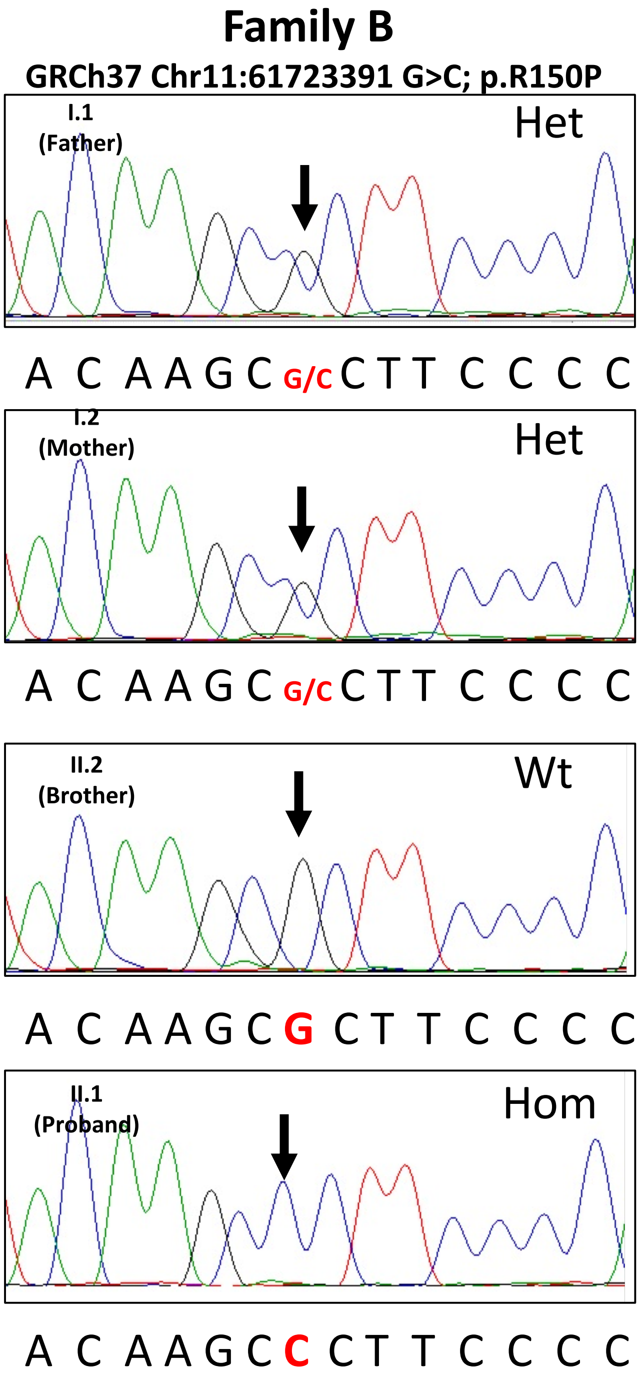

Supplementary Figure S9

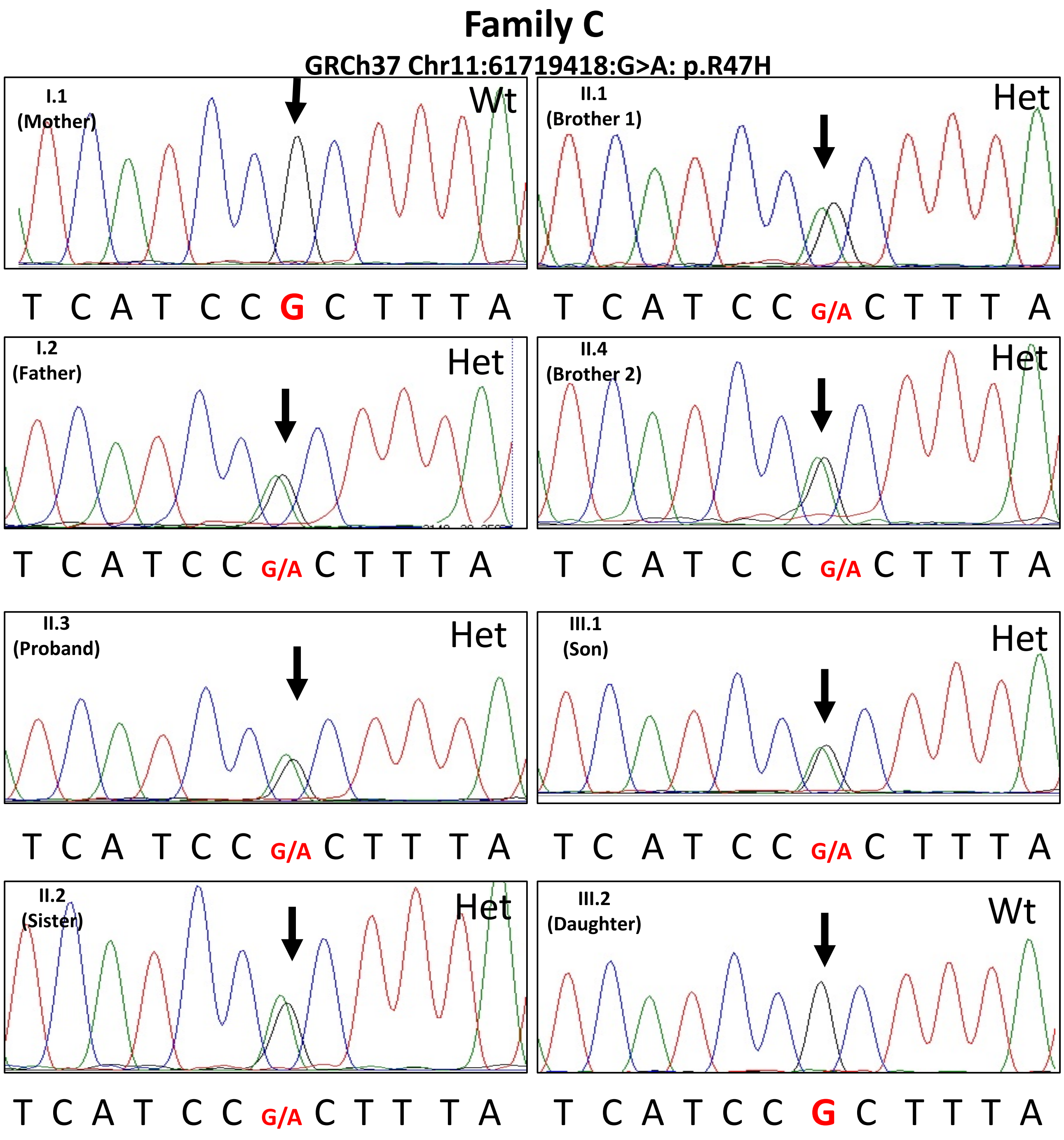

Supplementary Figure S10

Family C

GRCh37 Chr11:61725867:G>A; p.V216I

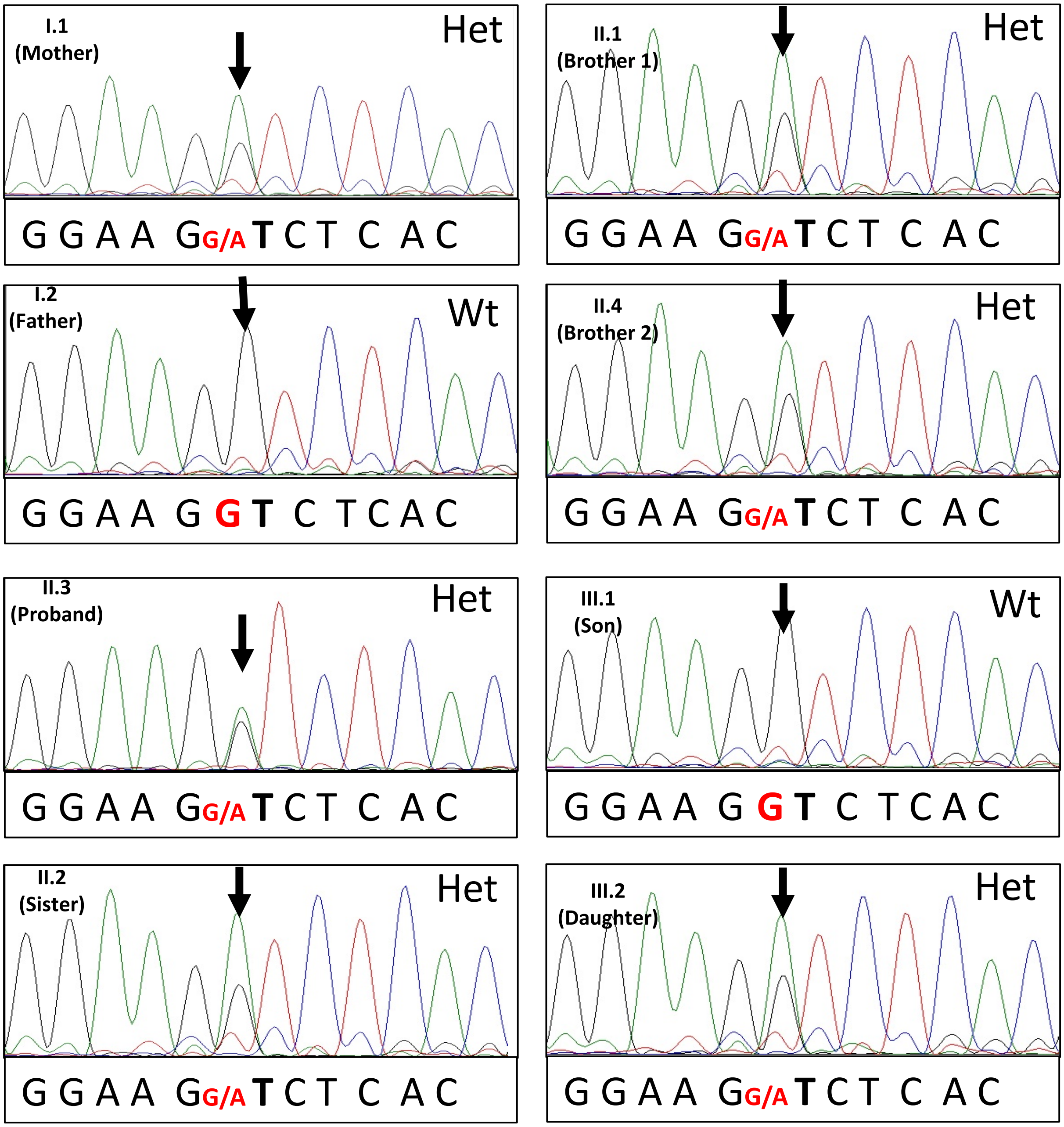

Supplementary Figure S11

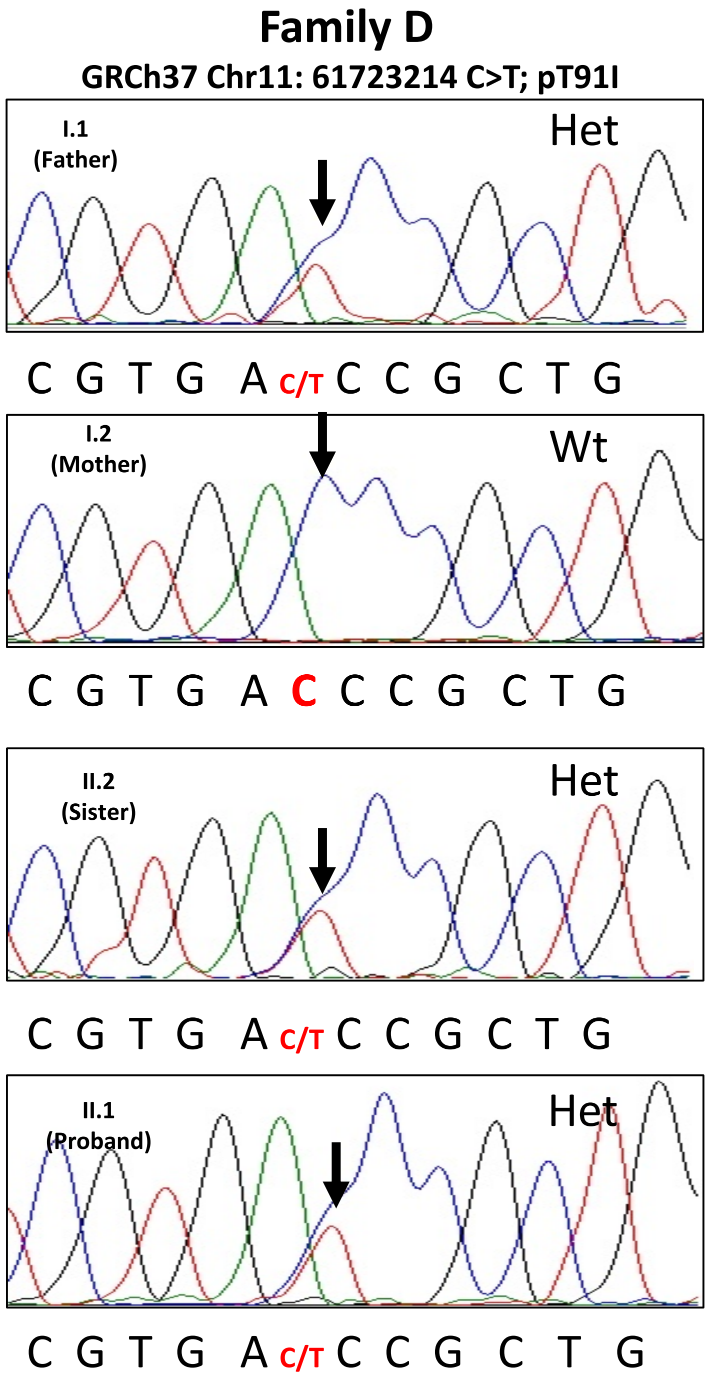

Supplement: Supplementary file 1 — Supplementary Figures S1-S11 [file 41598_2018_27951_MOESM1_ESM.pdf]
